# Synthesis of 2-aryl-5-(arylsulfonyl)-1,3,4-oxadiazoles as potent antibacterial and antioxidant agents

**DOI:** 10.55730/1300-0527.3366

**Published:** 2022-01-13

**Authors:** BODDAPATI S. N. Murthy, Subrahmanyam TALARI, A. Emmanuel KOLA, Bhuvaneswari CHALAPAKA

**Affiliations:** Department of Chemistry, Sir C R Reddy Autonomous College, Eluru, India

**Keywords:** 1, 3, 4-oxadiazole, antibacterial activity, antioxidant activity, DPPH, structure-activity relationship

## Abstract

Ten novel 2-aryl-5-(arylsulfonyl)-1,3,4-oxadiazoles were produced and assessed for their *in vitro* antibacterial and antioxidant activities. Diverse spectroscopic methods like ^1^H NMR, ^13^C NMR, IR, and LCMS were used for the characterization of the prepared samples and all the data was in good agreement with the anticipated structures. The prepared compounds **6a–j** were screened for their in vitro antibacterial activity against bacterial strains *Pseudomonas aeruginosa*, *Enterobacter aerogenes*, *Escherichia coli* (gram-positive), and *Bacillus cerus*, *Staphylococcus aureus*, *Bacillus subtilis* (gram-negative). The antimicrobial screening outcome revealed that the prepared 2-(3,4-dimethylphenyl)-5-tosyl-1,3,4-oxadiazole (**6j**), 2-(3-isopropylphenyl)-5-tosyl-1,3,4-oxadiazole (**6c**), and 2-(2-ethylphenyl)-5-tosyl-1,3,4-oxadiazole (**6i**) are most potent among all the examined compounds. Furthermore, the antioxidant activity of the prepared compounds was also investigated by DPPH radical scavenging method and the results showed that some of the compounds were moderately active.

## 1. Introduction

Heterocyclic compounds are a vital part of most of the bioactive molecules used as drugs and are the key motifs for the novel drug discovery. The heterocyclic compounds enhance their activity when fused with other ring systems [1–2]. Oxadiazoles with plethora of biological applications are identified as important construction motifs for the advance of innovative drug design [3–5], thus grabbing the attention of medicinal chemists around the world. With its capability to bind with a ligand, the oxadiazole ring can be used as a significant part of the pharmacophore. In certain instances, it behaves like a flat aromatic linker that affords the proper orientation of the molecule [6]. In the oxadiazole family, 1,3,4-oxadiazoles occupied a unique position in medicinal chemistry due to their multi-purpose utility in designing many bioactive compounds. In medicinal chemistry 1,3,4-oxadiazole and its derivatives are playing a vital role with broad range of biological applications. Oxadiazoles are the bioisostere of compounds with carbonyl function, like carboxylic acids, amides, and esters capable to form superior hydrogen bonding interactions with various receptors thereby augmenting the biological responses to a notable extent [7–8].

**Figure 1:**
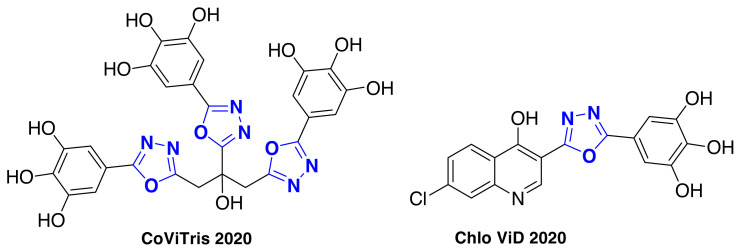
Chemical structures of CoViTris2020 and ChloViD2020 [9].

Recently, A.M. Rabie reported [9] two antioxidant polyphenolic 1,3,4-oxadiazole motifs, 2,3-tris[5-(3,4,5-trihydroxyphenyl)-1,3,4-oxadiazol-2-yl]propan-2-ol (CoViTris2020) and 5-[5-(7-chloro-4-hydroxyquinolin-3-yl)-1,3,4-oxadiazol-2-yl]benzene-1,2,3-triol (ChloViD2020) as the first multi-target SARS-CoV-2 inhibitors (Figure 1), with greater potency than the currently used medicine ivermectin, remdesivir, and favipiravir. The computational docking investigation of these two compounds displayed incredible high inhibitory binding affinities with most of the docked SARS-CoV-2/human proteins. Interestingly, the results of the biological assay showed that CoViTris2020 and ChloViD2020 exhibited very high and extremely significant anti-COVID-19 activities (anti-SARS-CoV-2 EC_50_ = 0.31 and 1.01 μM, respectively), representing that they can be very promising parent lead compounds for the design and construction of novel anti-COVID-19 agents.

**Figure 2:**
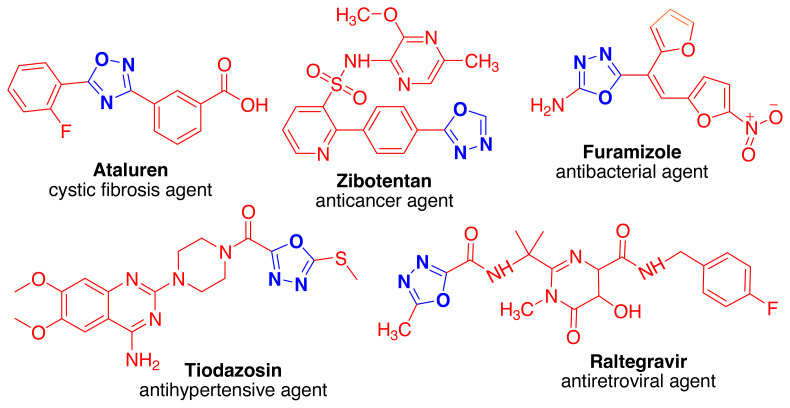
Drugs with 1,3,4-oxadiazole nucleus [28–29].

Moreover, 1,3,4-oxadiazoles have engrossed the attention of medicinal chemists as serotonin receptor (5-HT_3_) antagonists [10], Human Neurokinin-1 (NK_1_) antagonists [11], benzodiazepine receptor agonists [12], muscarinic agonists[13], **5**-hydroxytryptamine (5-HT_1D_) receptor agonists [14], antirhinoviral [15], tyrosinase inhibitory compounds[16]. Among the 1,3,4-oxadiazole family 1,3,4-oxadiazoles with substitutions at 2^nd^ and 5^th^ positions are an imperative core of bioactive molecules. These scaffolds are notorious for a variety of pharmacological activities like as antiinflammatory and analgesic [17], antibacterial [18], anticancer [19], antiviral [20], antihypertensive [21], herbicidal [22], antidiarrheal [23], monoamine oxidase (MAO) inhibitor [24], anti-HIV [25], anticonvulsant, and sedative hypnotic activity [26] hypoglycemic activity [27]. Some commercially marketed prominent clinical drugs like Atalurencystic-fibrosis agent, Furamizole-Zibotentan-anticancer agent, Tiodazosin-antihypertensive agent, and Raltegravir-antiretroviral agent (Figure 2) contain 1,3,4-oxadiazole units [28–29].

The construction of valuable 2,5-disubstituted-1,3,4-oxadiazoles was achieved by various methods such as 2-Iodoxybenzoic acid (IBX)/tetraethylammonium bromide (TEAB) [30], Fe(III)/2,2,6,6-Tetramethylpiperidin-1-yl) oxyl (TEMPO) [31], Cu(OTf)_2_ [32], molecular I_2_ [33] catalyzed oxidative cyclization of aroyl/acyl hydrazones, one-pot reaction of diverse aryl carboxylic acids and benzoyl hydrazides using alumina [34], Ph_3_P-I_2_ mediated dehydrative cyclization of *N*-acylbenzotriazoles and ethyl carbazate [35], I_2_ mediated oxidative C–O/C–S bond formation of semicarbazones [36], tosyl chloride/pyridine-mediated cyclization of thiosemicarbazides [37], oxidative annulation of *N*-acyl hydrazines [38].

Recently we have reported some efficient methodologies towards the construction of medicinally important heterocycles [39–40]. Moreover, in continuation of our efforts towards the development of therapeutically important heterocyclic compounds [41–44], and in view of plethora of bioapplications of 2,5-disubstituted-1,3,4-oxadiazole motifs herein we wish to report the construction of various 2-aryl-5-(arylsulfonyl)-1,3,4-oxadiazoles (Scheme). Moreover, the prepared compounds are evaluated for their antibacterial and antioxidant properties.

## 2. Experimental

### 2.1. Materials and methods

All the chemicals and reagents were acquired from Aldrich, Merck and utilized with no extra purification. After purchase, the solvents are dried prior to use by standard procedure [45]. The melting point of all the prepared compounds was recorded using the Cintex melting point apparatus in open capillaries. Precoated thin layer chromatography (TLC) plates (0.25 mm, Merck, silica gel 60 F_254_) were utilized to monitor all the reactions. A Varian-400 spectrometer was used to record the NMR (400MHz) spectra. All our experimental procedures were carried out by using a Centrifuge machine (VKSI-Medico) for the construction of the titled compounds.

### 2.2. General procedure for the synthesis of 2-aryl-1,3,4-oxadiazoles

The preparation of 2-(4-methylphenyl)-1,3,4-oxadiazole (**3a**) is exemplary for the construction of titled 2-aryl-5-(arylsulfonyl)-1,3,4-oxadiazoles. In a dried 100 mL two-necked reaction flask 30 mL of dry DMF, 3.5 g of 4-methylbenzoic acid (25.0 mmol) and 4.2 g of K_2_CO_3_ (30.0 mmol) were added and stirred well. Next, to this stirred solution, methyl iodide (2.5 mL, 30.0 mmol) was added dropwise for 10 min. The total mixture was stirred for 10 h at ambient temperature. Then the crude mixture was poured into ice water and extracted with hexane/EtOAc (20:5, v/v). After that, the organic layer was evaporated by utilizing a rotator evaporator. Silica gel (60–120 mesh) column chromatography was employed to purify the product with hexane/ethyl acetate (10:2, v/v) as eluent giving 4-methylbenzoate (3.4 g) in quantitative yield. Further, the obtained 4-methylbenzoate (3.4 g, 25.0 mmol), hydrazine monohydrate (7.5 g, 150.0 mmol), and EtOH (25 mL) were placed in a round bottom flask equipped with a condenser. The total mixture was stirred at reflux for 8 h and the reaction mixture was cooled to room temperature. Next, the mixture was concentrated using a rotary evaporator. The resulting residue was filtered with hexane and dried, affording 2.8 g of benzhydrazide **2a** as the product in 90% yield [46].

### 2.3. Preparation of 2-(p-tolyl)-5-tosyl-1,3,4-oxadiazole (6a)

In a 200 mL two-necked flask with triethyl orthoformate (TEOF) (25 mL), benzhydrazide **2a** (4 g, 27.0 mmol) was added and stirred vigorously at 140 °C for 5 h. At reduced pressure, the formed ethanol and residual triethyl orthoformate were distilled off. The residue on distillation under high vacuum (about 0.2 mbar) gave the product 2-*p*-tolyl-1,3,4-oxadiazole (**3a)** in 89% yield [47]. Next, the obtained 2-*p*-tolyl-1,3,4-oxadiazole (**3a**, 0.5 mmol) was treated with 4-methylbenzenethiol (**4a**) (1.25 mmol), followed by the addition of FeCl_3_ (1.25 mmol) and K_2_CO_3_ (2 mmol) in DMSO (5 mL). Then the total mixture was stirred at 50 °C for 15 h, to obtain the product 2-(*p*-tolylthio)-5-p-tolyl-1,3,4-oxadiazole **5a** in 56% yield. Furthermore, the intermediate **5a** (1 mmol) was oxidized with mCPBA in the presence of DCM as a solvent at ambient temperature for 3h to give 2-(p-tolyl)-5-tosyl-1,3,4-oxadiazole **6a**. The remaining final compounds also have been prepared in the same procedure.

#### 2.3.1. 2-(p-tolyl)-5-tosyl-1,3,4-oxadiazole (6a)

Yield (80%); White solid, mp. 85–87 °C; IR (KBr, υmax, cm^−1^): 2990 (Ar = CH str), 2896 (CH str), 1600, 1545, 1497 (Ar C = C str), 1449 (C = N str), 1261(N-N str), 1167 (C-O str); ^1^H NMR (400 MHz, CDCl_3_) δ ppm: ^1^H NMR (400 MHz, CDCl_3_) 8.29–8.20 (m, 2H), 7.67–7.62 (d, *J* = 7 Hz, 1H), 7.52–7.49 (m, 3H), 7.37–7.24 (t, *J* = 0.7 Hz, 1H), 7.17–7.12 (dd, *J* = 8.5, 0.7 Hz, 1H), 2.48 (s, 3H), 2.15 (s, 3H); ^13^C NMR (100MHz CDCl_3_) 163.0, 151.4, 140.04, 135.7, 131.2, 129.1, 128.0, 127.5, 125.7, 119.6, 24.5, 21.6; Elemental Analysis: Anal. Calcd. for C_16_H_14_N_2_O_3_S: C, 61.13; H, 4.49; N, 8.91; S, 10.20; Found: C, 61.44; H, 4.43; N, 8.79; S, 10.12. LC-MS (*m/z*): 315.32 (M+1)^+^.

#### 2.3.2. 2-(4-methoxyphenyl)-5-tosyl-1,3,4-oxadiazole (6b)

Yield (84%); White solid, mp. 70–72 °C; IR (KBr, υ_max_, cm^−1^): 3055 (Ar C-H str), 2926 (C-H str), 1582, 1486 (ArC = C str), 1439 (C = N str), 1386, 1372, and 1179, 1138 (C—C(CH_3_)_2_ str), 1374(C = N str), 1168 (C-O-C str); ^1^H NMR (400 MHz, CDCl_3_) δ 7.47–7.40 (m, 3H), 7.29 (s, 1H), 7.17–7.10 (m, 4H), 3.84 (s, 3H), 2.29 (s, 3H); ^13^C NMR (100 MHz, DMSO-d_6_ +CDCl_3_) δ 154.9, 153.1, 142.7, 133.8, 132.5, 129.8, 129.6, 121.3, 114.7, 55.4, 20.5; Elemental Analysis: Anal. Calcd. for C_16_H_14_N_2_O_4_S; C, 58.17; H, 4.27; N, 8.48; S, 9.71; Found: C, 58.47; H, 4.08; N, 8.17; S, 9.41; LC-MS (*m/z*): 331.21 (M+1)^+^.

#### 2.3.3. 2-(3-isopropylphenyl)-5-tosyl-1,3,4-oxadiazole (6c)

Yield (85 %); Yelllow solid, mp. 103–104 °C; IR (KBr, υ_max_, cm^−1^): 2932 (Ar = C-H str), 2836 (C-H str), 1649, 1530, 1488 (ArC = C str), 1386(C = N str), 1210 (N-N str); ^1^H NMR (400 MHz, CDCl_3_) δ 7.64–7.41 (m, 3H), 7.38–7.21 (m, 2H), 7.17 (d, *J* = 8.3 Hz, 3H), 2.89–2.84 (m, 1H), 2.44 (s, 3H), 1.22 (d, *J* = 6.7 Hz, 6H); ^13^C NMR (100 MHz, DMSO-d_6_+CDCl_3_) δ 151.6, 141.8, 141.2, 136. 0, 132.4, 128.5, 128.2, 128.0, 125.1, 120.2, 117.3, 115.4, 31.9, 22.5, 19.3; Elemental Analysis: Anal. Calcd. for C_18_H_18_N_2_O_3_S: C, 59.46; H, 4.99; N, 12.24; S, 9.34; Found: C, 59.76; H, 4.91; N, 12.17; S, 9.10; LC-MS (*m/z*): 343.25 (M+1)^+^.

#### 2.3.4. 2-(4-nitrophenyl)-5-tosyl-1,3,4-oxadiazole (6d)

Yield (68%); White solid, mp. 116–118 °C, IR (KBr, cm^−1^): IR (KBr, υ_max_, cm^−1^): 3136 (Ar=C-H str), 2948, 2872 (C-H str), 1592, 1449, 1439 (ArC = C str), 1534 (N-O str), 1348 (N-O str), 1189 (N-N str); ^1^H NMR (400 MHz, CDCl_3_) 8.25–8.20 (m, 3H), 7.53–7.48 (m, 3H), 7.24–7.06 (m, *J* = 3 Hz, 2H), 2.49 (s, 3H); ^13^C NMR (CDCl_3_, 100 MHz) 162.6, 158.9, 147.1, 142.6, 136.0, 131.7, 129.1, 127.9, 126.8, 123.7, 23.2; Elemental Analysis: Anal. Calcd. for C_15_H_11_N_3_O_5_S: C, 52.17; H, 3.01; F, 15.47; N, 7.61; O, 13.03; S, 8.71; Found: C, 52.44; H, 3.78; N, 7.53; S, 8.62; LC-MS (*m/z*): 346.21 (M+1)^+^.

#### 2.3.5. 3-(5-tosyl-1,3,4-oxadiazol-2-yl)benzonitrile (6e)

Yield (65 %); White solid, mp. 70–71 °C; IR (KBr, υ_max_, cm^−1^): 3064 (Ar = C-H str), 2922 (C-H str), 2224 (CN Str). 1601, 1479, 1450 (ArC = C str), 1400 (C = N str), 1262 (N-N str); ^1^H NMR (400 MHz, DMSO) δ 7.69 (d, *J* = 7.5 Hz, 2H), 7.46 (d, J=7.7 Hz, 2H), 7.38 (d, *J* = 8.3 Hz, 2H), 7.16–7.00 (d, *J* = 8.6 Hz, 2H), 2.38 (s, 3H); ^13^C NMR (100 MHz, CDCl_3_ + DMSO-d_6_) δ 165.8, 162.9, 143.6, 143.1, 139.0, 133.8, 132.7, 129.4, 128.9, 121.5, 117.9, 117.5, 114.7, 20.9; Elemental Analysis: Anal. Calcd. for C_16_H_11_N_3_O_3_S: C, 55.80; H, 3.68; N, 13.95; O, 15.93; S, 10.64; Found: C, 56.08; H, 3.65; N, 13.87; S, 10.52; LC-MS (*m/z*): 326.25 (M+1)^+^.

#### 2.3.6. 2-(4-chlorophenyl)-5-tosyl-1,3,4-oxadiazole (6f)

Yield (74%); White solid, mp. 119–120 °C; IR (KBr, υ_max_, cm^−1^): 3098 (Ar = C-H str), 2960, 2920 (C-H str), 1607, 1530, 1478 (ArC = C str), 1350 (C = N str), 1238 (N-N str), 766 (C-F str); ^1^H NMR (400 MHz, CDCl_3_) δ 7.66 (d, 2H), 7.48 (t, *J* = 9.4 Hz, 2H), 7.27 (d, *J* = 7.5 Hz 2H), 7.24 (s, 1H), 7.17 (d, *J* = 8.7 Hz, 2H), 2.33 (s, 3H); ^13^C NMR (100 MHz, CDCl_3_ + DMSO-d_6_) δ 151.2, 141.8, 137.0, 132.4, 128.3, 128.2, 127.8, 126.9, 119.9, 118.2, 20.0; Elemental Analysis: Anal. Calcd. for C_15_H_11_ClN_2_O_3_S: C, 53.82; H, 3.31; Cl, 10.59; N, 8.37; S, 9.58; Found: C, 54.09; H, 3.27; N, 8.31; S, 9.82; LC-MS (*m/z*): 317.21 (M+1)^+^.

#### 2.3.7. 2-(4-fluorophenyl)sulfonyl)-5-phenyl-1,3,4-oxadiazole (6g)

Yield (72%); White solid, mp. 96–97 °C; IR (KBr, υ_max_, cm^−1^): 3095 (Ar = C-H str), 2955, 2921, 2863 (C-H str), 1626, 1583, 1494 (ArC = C str), 1383 (C = N str), 1233 (N-N str), 829 (C-Fstr); ^1^H NMR (400 MHz, CDCl_3_) δ 7.39 (d, *J* = 4.7 Hz, 2H), 7.31–7.21 (m, 3H), 7.11 (s, 1H), 7.04 (d, *J* = 6.3 Hz, 3H); ^13^C NMR (100 MHz, CDCl_3_) δ 164.6, 160.9, 146.9, 136.5, 128.9, 127.0, 125.4, 123.6, 119.3, 116.2; Elemental Analysis: Anal. Calcd. for C_14_H_9_FN_2_O_3_S: C, 55.26; H, 2.98; F, 6.24; N, 9.21; S, 10.54; Found: C, 54.53; H, 2.93; N, 9.14; S, 10.11; LC-MS (*m/z*): 305.13 (M+1)^+^.

#### 2.3.8. 2-((4-methoxyphenyl)sulfonyl)-5-phenyl-1,3,4-oxadiazole (6h)

Yield (81%); White solid, mp. 92–93 °C; IR (KBr, υmax, cm^−1^): 3130 (Ar = CH str), 2955 (C-H str), 1614, 1519, 1459 (ArC = C str), 1372 (C = N str), 1278 (N-N str), 1172 (C-O-C str); ^1^H NMR (400 MHz, CDCl_3_) δ 7.78 (d, *J* = 7.7 Hz, 2H), 7.56–7.48 (m, 3H), 7.41 (d, *J* = 8.7 Hz, 2H), 6.85 (d, *J* = 8.7 Hz, 2H), 3.80 (s, 3H); ^13^C NMR (100 MHz, DMSO-d_6_ +CDCl_3_) δ 155.1, 153.1, 133.6, 132.2, 131.9, 129.6, 128.6, 120.3, 114.4, 113.5, 55.2; Elemental Analysis: Anal. Calcd. for C_15_H_12_N_2_O_4_S: C, 56.95; H, 3.82; N, 8.86; S, 10.14; Found: C, 57.12; H, 3.78; N, 8.69; S, 9.87; LC-MS (*m/z*): 317.19 (M+1)^+^.

#### 2.3.9. 2-(2-ethylphenyl)-5-tosyl-1,3,4-oxadiazole (6i)

Yield (81%); White solid, mp. 98–99 °C; IR (KBr, υ_max_, cm^−1^): IR (KBr, υ_max_, cm^−1^): 3066 (Ar C-H str), 2956, 2932 (C-H str), 1589, 1560, 1512(ArC = C str), 1340 (C = N str), 1216 (N-N str); ^1^H-NMR (400 MHz, CDCl_3_) δ 7.64 (d, 2H), 7.41 (d, *J* = 8.5 Hz, 2H), 7.38–7.34 (m, 2H), 7.14 (d, *J* = 8.2 Hz, 2H), 2.62–2.58 (q, 2H), 2.45 (s, 3H), 1.16 (t, *J* = 7.7 Hz, 3H); ^13^C NMR (100 MHz, DMSO-d_6_ + CDCl_3_) δ 163.7, 151.5, 141.6, 136.2, 135.7, 132.3, 128.2, 128.0, 126.4, 122.7, 120.1, 117.0, 26.2, 21.4, 19.2; Elemental Analysis: Anal. Calcd. for C_17_H_16_N_2_O_3_S: C, 58.17; H, 4.27; N, 8.48; S, 9.71; Found: C, 58.36; H, 4.75; N, 8.38; S, 9.59; LC-MS (*m/z*): 329.27 (M+1)^+^.

#### 2.3.10. 2-(3,4-dimethylphenyl)-5-tosyl-1,3,4-oxadiazole (6j)

Yield (84%); White solid, mp. 61–62 °C; IR (KBr, υ_max_, cm^−1^): IR (KBr, υ_max_, cm^−1^): 3032 (ArC-H str), 2926 (C-H str),1585, 1548, 1492(ArC = C str), 1374 (C = N str), 1212 (N-N str); ^1^H NMR (400 MHz, CDCl_3_) δ 7.79 (d, 2H), 7.66 (d, 2H), 7.37–7.34 (m, 2H), 7.01 (d, *J* = 7.5 Hz, 1H), 6.83 (d, *J* = 7.3 Hz, 1H), 2.46 (s, 3H), 2.33 (s, 3H), 2.12 (s, 3H); ^13^C NMR (100 MHz, CDCl_3_ + DMSO-d_6_) δ 164.3, 162.7, 136.8, 135.3, 133.6, 130.2, 129.5, 129.3, 129.2, 126.0, 125.1, 121.1, 22.3, 20.5, 20.4; Elemental Analysis: Anal. Calcd. for C_17_H_16_N_2_O_3_S: C, 61.13; H, 4.49; N, 8.91; S, 10.20; Found: C, 61.45; H, 4.44; N, 8.83; S, 10.09; LC-MS (*m/z*): 329.30 (M+1)^+^.

### 2.4. Procedure for antibacterial activity [48]

DMSO solution of all the prepared compounds at a concentration of 1mg/mL was prepared individually. In sterile Mueller Hinton medium each bacterium was inoculated and kept at 37 °C for 24 h to develop inoculums. The bacterial suspension was diluted by utilizing sterile saline to regulate the turbidity to the 0.5 McFarland standards. Next, on sterile Mueller Hinton agar plates, diluted suspension (200 μL) of every pathogen was inoculated. Wells were punched in the agar medium. Next, 100 μL of every compound solution was placed in a separate well with a micropipette. In addition, to check the activity of DMSO against the pathogenic culture, 100 μL of pure DMSO solution was also placed in a well and the entire petri dishes were incubated at 37 °C for 24 h. A clear zone around the well was regarded as positive results. The antimicrobial potency of the examined compounds was calculated after inclusive incubation. Finally, the zone of inhibition was calculated and recorded in millimetres (mm).

### 2.5. Procedure for antioxidant activity

Ai Lan Chew et al. method [49] was used to determine the 2,2-Diphenyl-1-picryl hydrazyl (DPPH) free radical scavenging activity of the various extracts. The crude extracts in diverse concentrations viz., 25 μg/mL, 50 μg/mL, 100 μg/mL, and 200 μg/mL were prepared in dimethyl sulphoxide (DMSO). 1 mL of every concentration was mixed with 4 mL of 0.004% (w/v) solution of DPPH prepared in CH_3_OH. The reaction mixture was set aside in dark for incubation for 30 min. CH_3_OH was used as control and ascorbic acid was employed as positive control. The absorbance was calculated at 517 nm. The following formula was used to determine the DPPH scavenging activity (%). DPPH scavenging activity (%) = [(AO–AS)/AO] × 100, where, AO = absorbance of the control, AS = absorbance of the plant sample.

## 3. Results and discussion

### 3.1. Chemistry

In current study, the authors illustrated the construction of novel 2-aryl-5-(arylsulfonyl)-1,3,4-oxadiazoles as presented in Scheme. Initially, diverse aryl acids were treated with hydrazine monohydride in presence of base K_2_CO_3_ to give the respective aryl hydrazides, which undergo cyclization in presence of triethylorthoformate (TEOF) in ethanol to form the corresponding 2-aryl-1,3,4-oxadiazoles in 56%–68% of yields. Next, the obtained 2-aryl-1,3,4-oxadiazoles were treated with various thiophenols in presence of FeCl_3_ using K_2_CO_3_ as a base in DMSO to give the respective *C-S* cross-coupled product 2-thioaryl-5-aryl-1,3,4-oxadiazoles **5a–j**. Finally, the compounds **5a–j** on oxidation with mCPBA in DCM yield the respective 2-aryl-5-(arylsulfonyl)-1,3,4-oxadiazoles **6a–j** in moderate to good yield (65%–85%).

**Figure 3:**
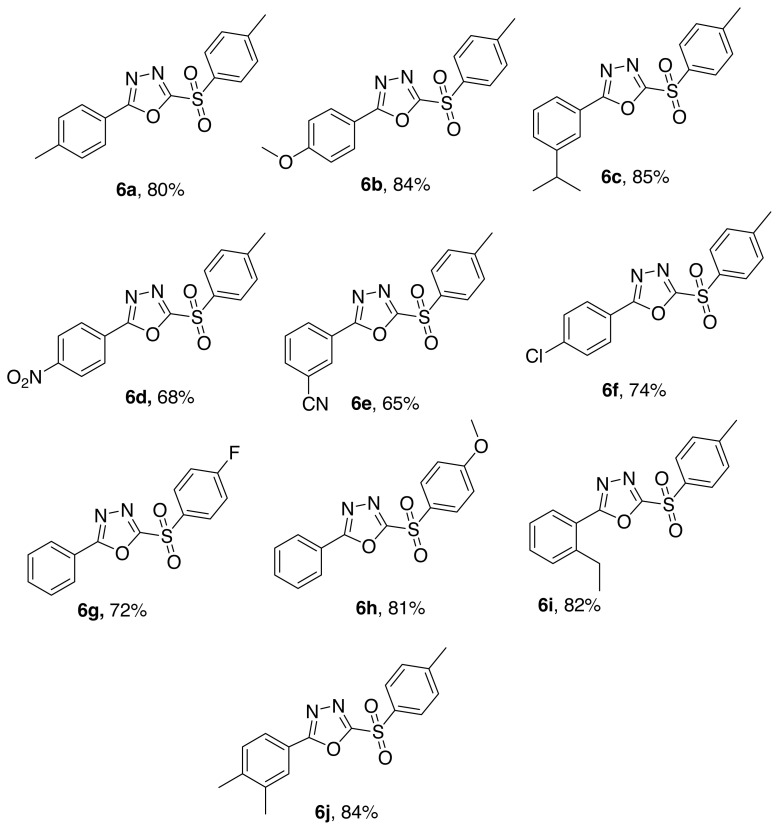
Synthesized final 1,3,4-oxadiazoles.

Various 2-aryl-5-(arylsulfonyl)-1,3,4-oxadiazoles (Figure 3) were obtained using diversely substituted aryl hydrazides and thio phenols. Compounds bearing phenyl ring with electron donating groups like –CH_3_, –OCH_3_, -Et, and -CH(CH_3_)_2_ obtained at higher yields contrast to those with electron withdrawing groups like –CN,–NO_2_ and weak electron withdrawing groups like fluorine and chlorine.

The structure of all the prepared compounds was attributed with IR, NMR (^1^H and ^13^C), and LC-MS spectral analyses and the spectroscopic and analytical data was in complete agreement with the anticipated structures. For example, in the IR spectrum of compound **6h**, the appearance of a peak at 2224 (s) cm^−1^ indicates the presence of –C ≡ N group, formation of distinguishing peaks at 3064 (w) cm^−1^, 1111 (s) cm^−1^ owing to Ar C-H, and C-O-C groups of oxadiazole frame. The appearance of IR peaks at 1400(s) cm^−1^ and 1262 (w) cm^−1^ are due to –C = N and N-N stretching’s. Next, IR peaks at 1607(m), 1569 (s), and 1531 (w) cm^−1^ are due to aromatic C = C stretching. Further, a peak of 2922(s) cm^−1^ characterize the –C-H stretching of CH_3_ group, asserted the formation of title compound **6h**.

Next, the emergence of a signal in the ^1^H-NMR spectrum of **6h** at chemical shift value *d* 3.80 ppm as a singlet, integrating for three protons were assigned to -O-CH_3_ group, doublets at *d* 7.78, 7.41 ppm integrating for two protons, multiplet at *d* 7.56–7.48 ppm integrating for three protons and another doublet at *d* 6.85 ppm integrating for two protons were assigned for aromatic protons. Moreover, the ^13^C-NMR spectrum of compound **6h** revealed the presence of 11 different carbons in the compound. The peak at *d* 55.0 ppm has been allocated to the methoxy carbon. The signals at *d* 155.1 and 153.1 ppm were due to the carbon of C-O core nuclei respectively. The signals at *d* 133.6–113.5 ppm have been consigned to the aromatic carbons of the compound. All the above spectral data indicate that compound **6h** is *2-((4-methoxyphenyl) sulfonyl)-5-phenyl-1,3,4-oxadiazole*. Moreover, the evolution of molecular ion peak at 317.19 (M+H)^+^ in the mass spectrum (EI) supported the formation of compound **6h**.

### 3.2. Biological evaluation

#### 3.2.1. Antimicrobial activity

The well diffusion method [48] was used to study the in vitro bacterial growth inhibition activity of the test compounds **6a–j** on gram-positive bacterial strains *Pseudomonas aeruginosa*, *Enterobacter aerogenes*, *Escherichia coli* and gram-negative bacterial strains *Bacillus cerus*, *Staphylococcus aureus*, *Bacillus subtilis*. The antibacterial activity screening outcome reveals that the compounds **6b, 6c, 6e, 6i**, and **6j** are active against all the six bacterial strains examined with a good zone of inhibition values (Table 1). The compounds **6j**, **6i**, and **6e** were found to inhibit the growth of *Pseudomonas aeruginosa* with Zone of inhibition values 22mm, 19mm, and 16mm respectively. The compounds **6j**, **6c**, **6e**, and **6f** showed good activity *against Enterobacter aerogenes* with a consecutive zone of inhibition of 23mm, 19mm, 18mm, and 18mm. Test compounds **6j** and **6i** with a zone of inhibition of 20 mm and 15 mm, effectively inhibit the growth of microorganism *Escherichia coli*. Compounds **6c** and **6j** exhibited good activity against the gram-negative organisms *Bacillus cerus*, *Staphylococcus aureus*, and *Bacillus subtilis* with a zone of inhibition range of 19–23 mm and 18–22 mm consecutively. In addition, the antibacterial screening (Table 1) discloses that the titled compounds are more potent against the gram-negative bacteria compared to gram-positive bacteria.

Based on the zone of inhibition values, next the minimum inhibitory concentration (MIC) value (mg/mL) was determined for the compounds that showed significant growth inhibition zones with the use of serial dilution method and the MIC values recorded in Table 2. The MIC results indicate that most of the tested compounds displayed variable inhibitory effects on the growth of tested bacterial strains. The MIC was deduced by following the method and guidelines of the Clinical and Laboratory Standard Institute (CLSI) (Table 2). In this study, the MIC was determined for the most potent selected antimicrobial compounds **6b, 6c**, **6e**, **6i**, and **6j**. The investigation reveals that the MIC value of test compounds is in the range of 132–98 μg/mL against *Enterobacter aerogenes* and 72–150 μg/mL against *Bacillus subtilis*. Among the test compounds, **6j** exhibited potent antibacterial activity with a minimum inhibitory concentration value of 98 μg/mL against *Enterobacter aerogenes*, and 72 μg/mL against *Bacillus subtilis*. Compound **6i** disclosed the minimum inhibitory concentration values of 103 μg/mL and 75 μg/mL against *Enterobacter aerogenes*, and against *Bacillus subtilis*. However, all the test compounds are less potent than the reference drug streptomycin.

#### 3.2.2. Antioxidant activity

The in vitro antioxidant activity of the prepared compounds **6a–6j** was evaluated by a standard literature protocol [49]. For this, different extracts were tested for their 2,2-diphenyl-1-picrylhydrazyl (DPPH) free radical scavenging activity according to the literature protocol. Dissimilar concentrations of the crude extracts with 25μg/mL, 50 μg/mL, 100 μg/mL, and 200 μg/mL concentrations were examined, by using ascorbic acid as a standard positive control. This investigation outcome is shown in Table 3.

The antioxidant activity screening outcomes (Table 3) reveal that all the prepared oxadiazole motifs showed good antioxidant activity. The title compounds **6a–6j** exhibited concentration reliant increase in antioxidant activity, i.e. their antioxidant activity was increased as the concentration increased. Compounds **6j** and **6b** displayed the highest and lowest antioxidant activities at the concentrations of 100 μg, 50 μg, and 25μg, respectively. But, compounds **6f** and **6a** exhibited utmost and least antioxidant activity respectively at the concentration of 200 μg. The compound **6c** displayed almost similar antioxidant activity at the concentration of 200 μg. All the remaining eight compounds except **6a** and **6b**, exhibited more than 40% level of antioxidant properties at 200 μg concentration. However, in comparison with standard ascorbic acid, all the prepared compounds displayed significantly lower antioxidant activity at all the tested concentrations.

From the results of antibacterial and antioxidant studies, it was assumed that (i) presence of electron donating functionality at 2^nd^ and 5^th^ position of benzene ring in **6j** is responsible for significant activity against the tested bacterial strains because the presence of +I effect groups in benzene ring system amplifies the lipophilicity and thus enhance cell penetration rate, that is accountable for antibacterial drug efficiency; (ii) the physicochemical characters such as position and kind of substituent on the aromatic ring of sulphoxide influence the antimicrobial activity of the examined compounds; (ii) presence of the electron donating groups are also responsible for their better antioxidant activities also; (iii) the electron withdrawing nitro group is responsible for the moderate antioxidant activity of compound **6d**.

## 4. Conclusion

A sequence of 2-aryl-5-(arylsulfonyl)-1,3,4-oxadiazole scaffolds were synthesized and evaluated for their antibacterial and antioxidant activities. The data obtained from spectroscopic techniques like IR, NMR, and LC-MS affirmed the structure of all the obtained compounds. The antimicrobial screening outcome of all these titled compounds revealed that the examined compounds **6j**, **6c**, and **6i** were the most potent among all prepared compounds. Moreover, the obtained compounds exhibited good antioxidant activity also. The target compounds **6j** and **6i** showed the highest antioxidant activity.

## Synthesis of 2-aryl-5-(arylsulfonyl)-1,3,4-oxadiazoles as potent antibacterial and antioxidant agents

^1^H-NMR spectrum of compound **6a**

^13^C-NMR spectrum of compound **6a**

Mass spectrum of compound **6a**

IR spectrum of compound **6a**

^1^H-NMR spectrum of compound **6b**

^13^C- NMR spectrum of compound **6b**

Mass spectrum of compound **6b**

IR spectrum of compound **6b**

^1^H-NMR spectrum of compound **6c**

^13^C-NMR spectrum of compound **6c**

Mass spectrum of compound **6c**

IR spectrum of compound **6c**

^1^H-NMR spectrum of compound **6d**

^13^C-NMR spectrum of compound **6d**

Mass spectrum of compound **6d**

IR spectrum of compound **6d**

^1^H-NMR spectrum of compound **6e**

^13^C-NMR spectrum of compound **6e**

Mass spectrum of compound **6e**

IR spectrum of compound **6e**

^1^H-NMR spectrum of compound **6f**

^13^C-NMR spectrum of compound **6f**

Mass spectrum of compound **6f**

IR spectrum of compound **6f**

^1^H-NMR spectrum of compound **6g**

^13^C-NMR spectrum of compound **6g**

Mass spectrum of compound **6g**

IR spectrum of compound **6g**

^1^H-NMR spectrum of compound **6h**

^13^C-NMR spectrum of compound **6h**

Mass spectrum of compound **6h**

IR spectrum of compound **6h**

^1^H-NMR spectrum of compound **6i**

^13^C-NMR spectrum of compound **6i**

Mass spectrum of compound **6i**

IR spectrum of compound **6i**

^1^H-NMR spectrum of compound **6j**

^13^C-NMR spectrum of compound **6j**

Mass spectrum of compound **6j**

IR spectrum of compound **6j**

## Figures and Tables

**Scheme:**
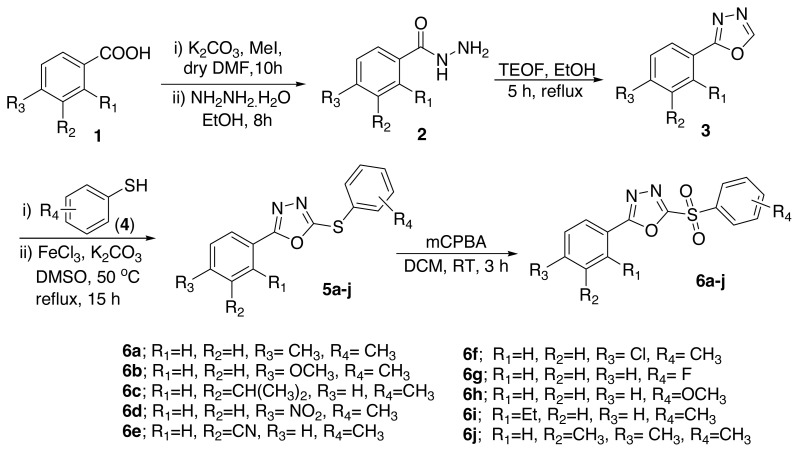
Synthesis of 2-aryl-5-(arylsulfonyl)-1,3,4-oxadiazoles (6a–j).

**Table 1:** Antibacterial activity in Zone of inhibition (mm) of the final compounds (**6a–6j**).

Compound	Diameter of Zone of Inhibition in mm					
	Microorganism					
	Gram −Ve			Gram +Ve		
	*PA*^a^ (−)	*EA*^b^(−)	*EC*^c^ (−)	*BC*^d^ (+)	*SA*^e^ (+)	*BS*^f^(+)
**6a**	11	13	-	16	-	14
**6b**	12	16	11	14	10	18
**6c**	10	19	12	23	13	19
**6d**	-	13	-	-	-	15
**6e**	16	18	11	15	14	20
**6f**	-	18	-	14	-	13
**6g**	-	17	10	-	11	-
**6h**	12	17	-	13	-	12
**6i**	19	17	15	16	11	19
**6j**	22	23	20	21	18	22
**Streptomycin (Standard)**	**32**	**33**	**29**	**33**	**29**	**32**

PA ^a^- Pseudomonas aeruginosa; EA^b^- Enterobacter aerogenes; EC^c^-Escherichia coli; BC^d^ - Bacillus cerus; SA^e^- Staphylococcus aureus; BS^f^- Bacillus subtilis; -: No inhibition.

**Table 2:** MIC values of most potent titled compounds (μg/mL).

Entry	E. aerogenes(−)	B. subtilis(+)
**6b**	114	150
**6c**	160	180
**6e**	132	120
**6i**	103	75
**6j**	98	72
**Streptomycin**	**30**	**25**

**Table 3:** Antioxidant activity of titled compounds **6a–j**.

Sample	Antioxidant activity (%)			
	25 μg	50 μg	100 μg	200 μg
**6a**	21.42	23.52	27.52	31.52
**6b**	16.78	21.67	24.67	33.67
**6c**	25.67	29.54	33.54	54.54
**6d**	31.12	35.73	42.13	46.13
**6e**	30.56	34.45	39.45	47.45
**6f**	31.19	35.19	40.19	55.19
**6g**	32.72	34.12	36.78	43.12
**6h**	27.34	32.34	37.34	40.34
**6i**	34.65	37.65	39.65	42.65
**6j**	40.23	45.45	47.78	48.46
**Ascorbic acid**	**78.74**	**86.06**	**92.81**	**93.25**
