# Supplementary figures and images for: Synthesis of 2-aryl-5-(arylsulfonyl)-1,3,4-oxadiazoles as potent antibacterial and antioxidant agents
Source: Turk J Chem. 2022 Jan 13;46(3):766–76. doi: 10.55730/1300-0527.3366 (PMC10503987; doi:10.55730/1300-0527.3366)

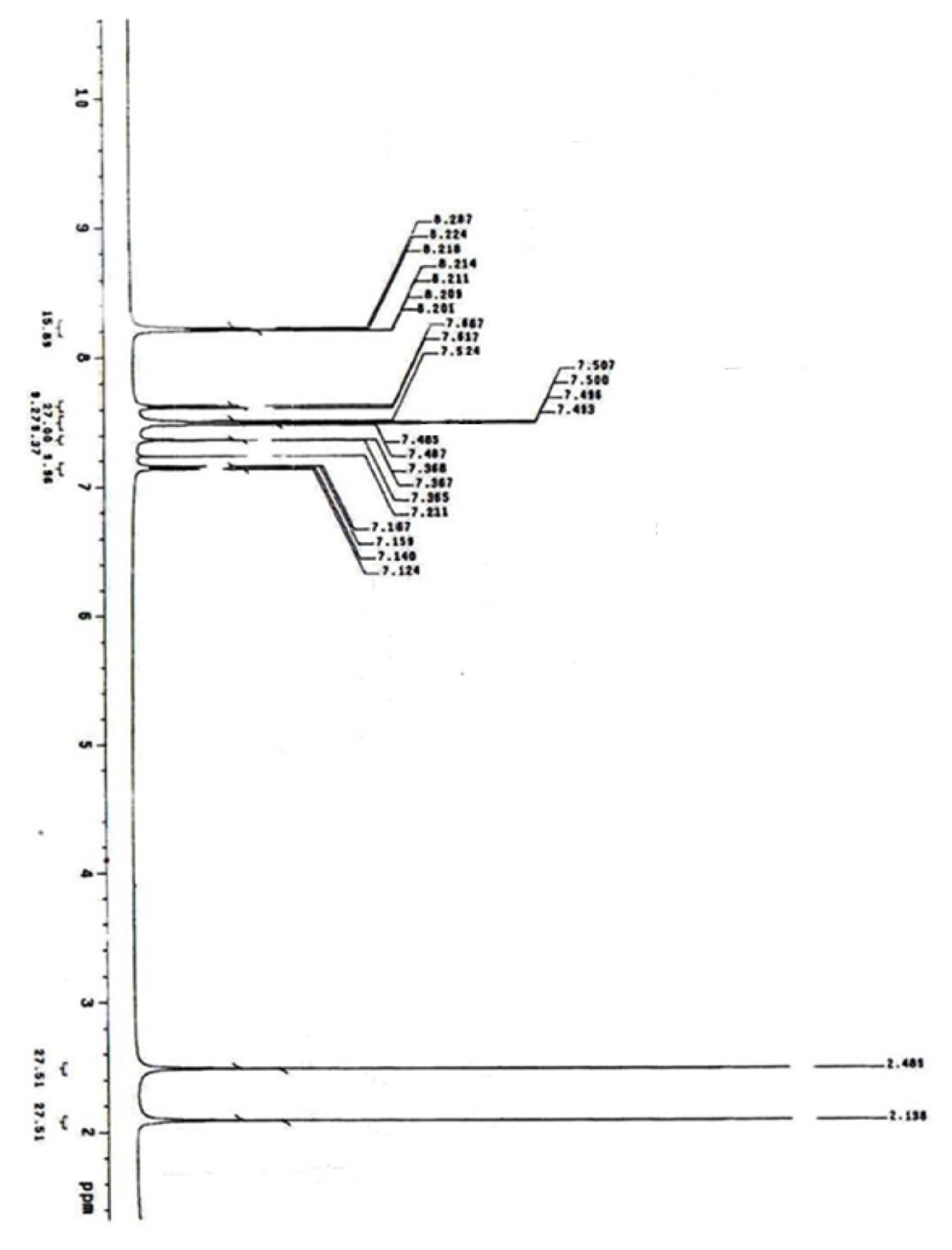

Supplement: Supplementary file 1 — 1H-NMR spectrum of compound 6a [file turkjchem-46-3-766s1.tif]

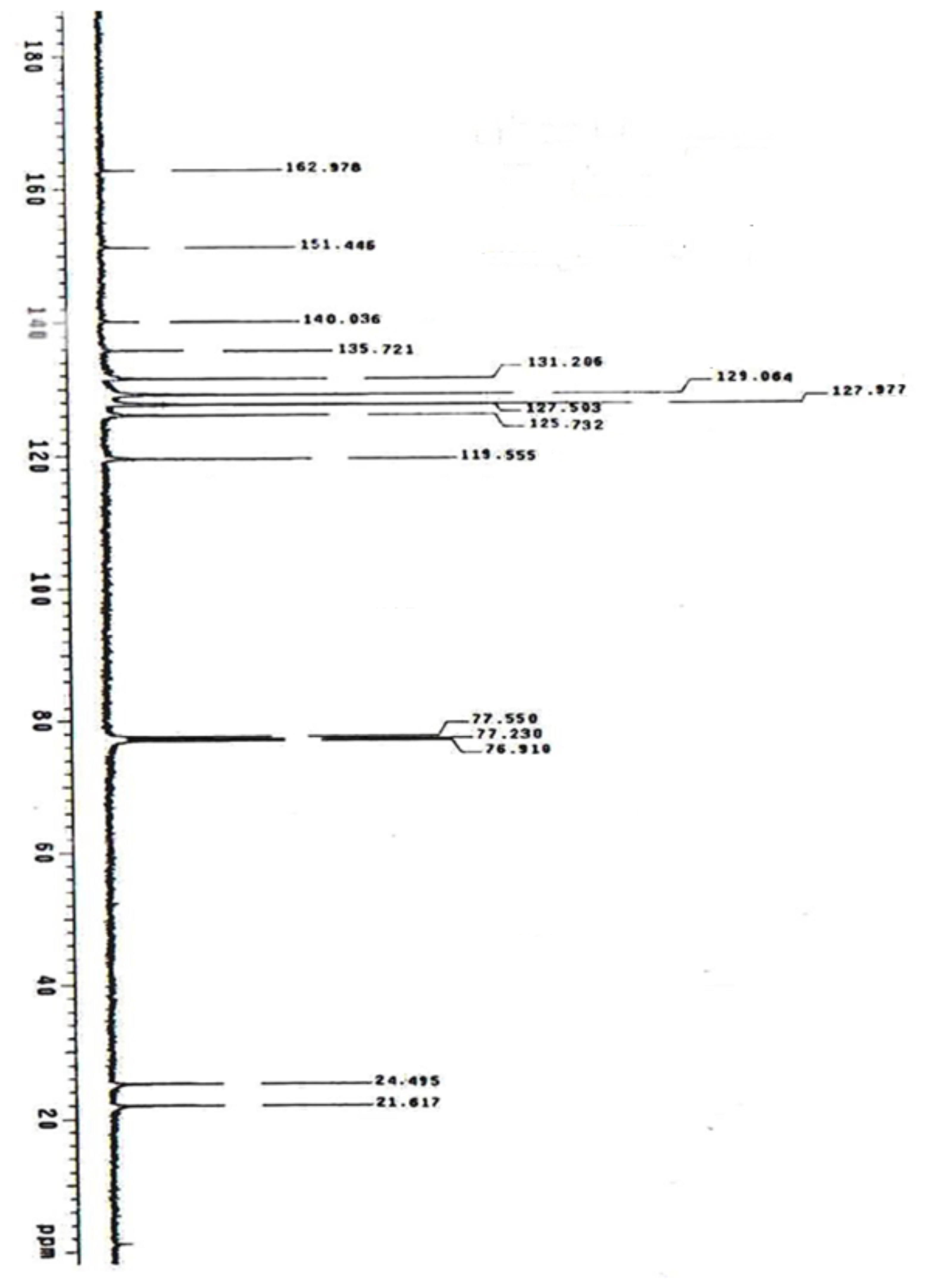

Supplement: Supplementary file 2 — 13C-NMR spectrum of compound 6a [file turkjchem-46-3-766s2.tif]

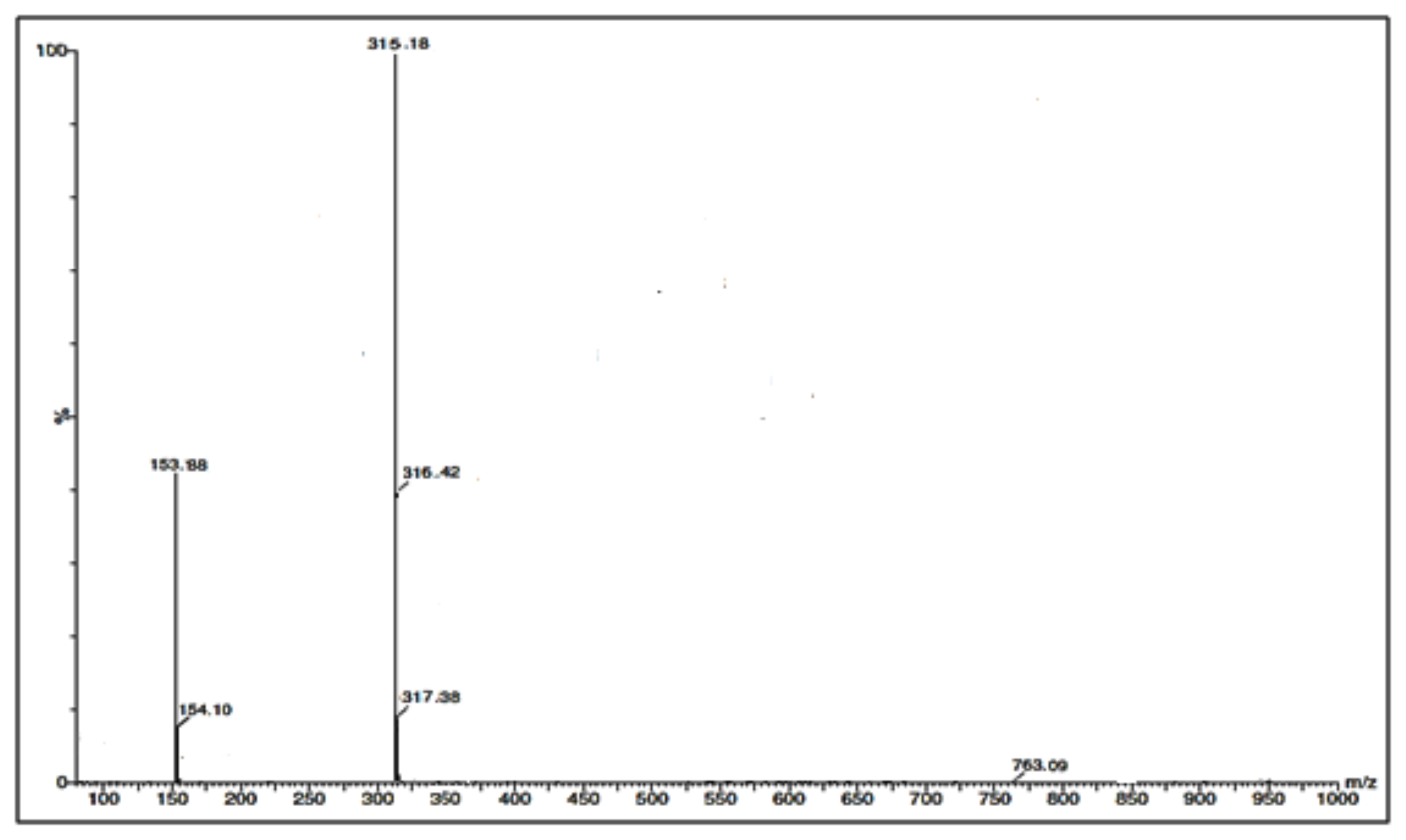

Supplement: Supplementary file 3 — Mass spectrum of compound 6a [file turkjchem-46-3-766s3.tif]

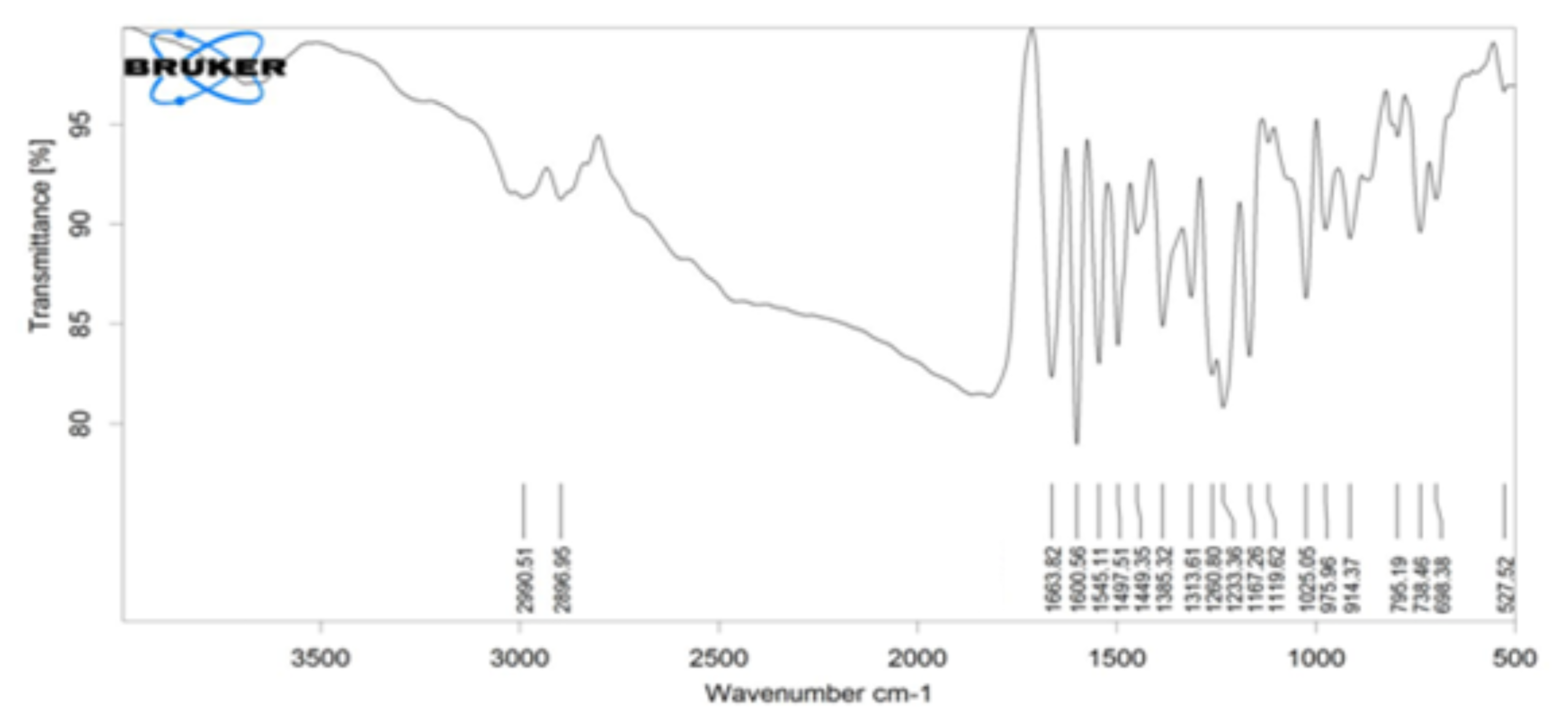

Supplement: Supplementary file 4 — IR spectrum of compound 6a [file turkjchem-46-3-766s4.tif]

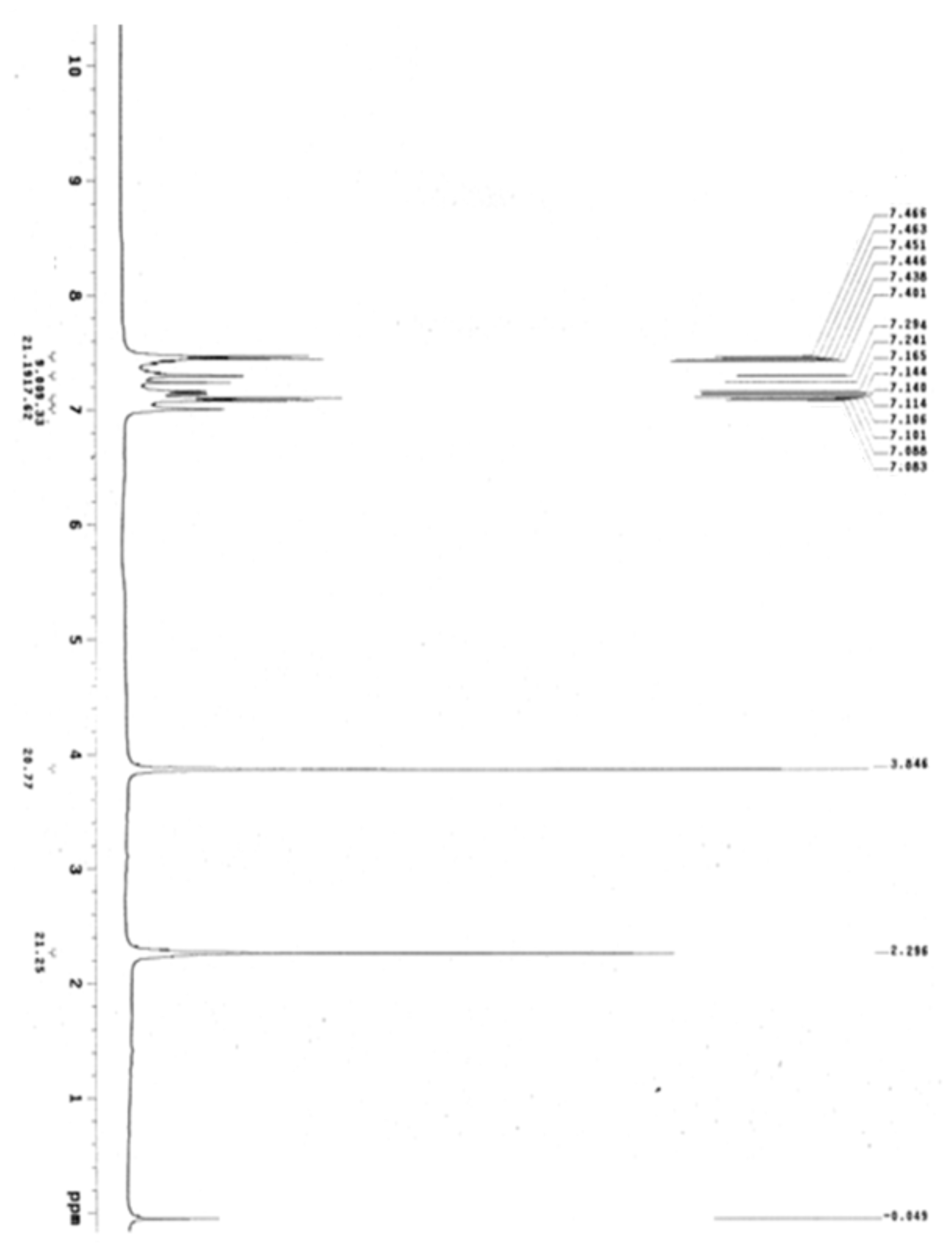

Supplement: Supplementary file 5 — 1H-NMR spectrum of compound 6b [file turkjchem-46-3-766s5.tif]

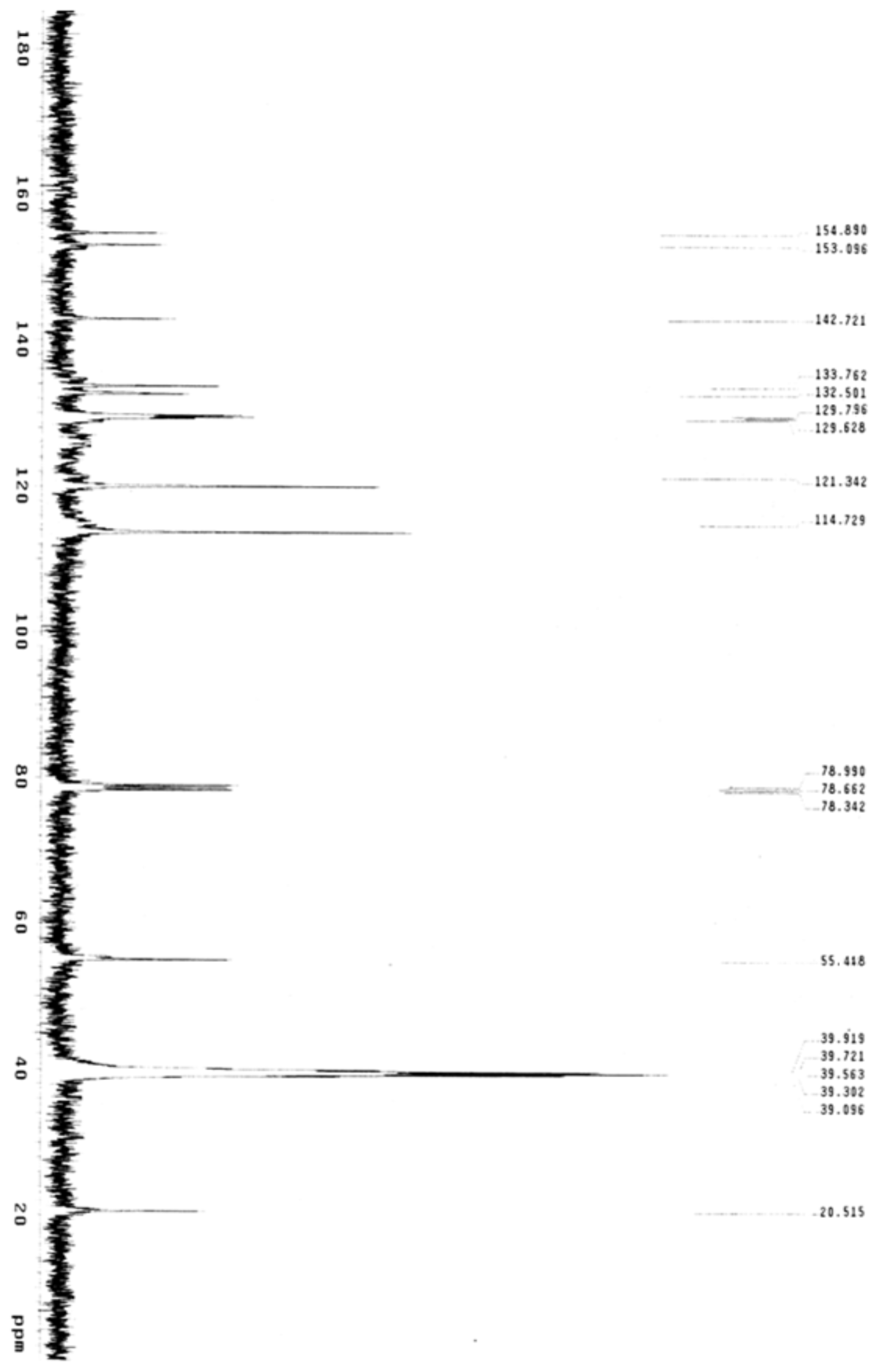

Supplement: Supplementary file 6 — 13C- NMR spectrum of compound 6b [file turkjchem-46-3-766s6.tif]

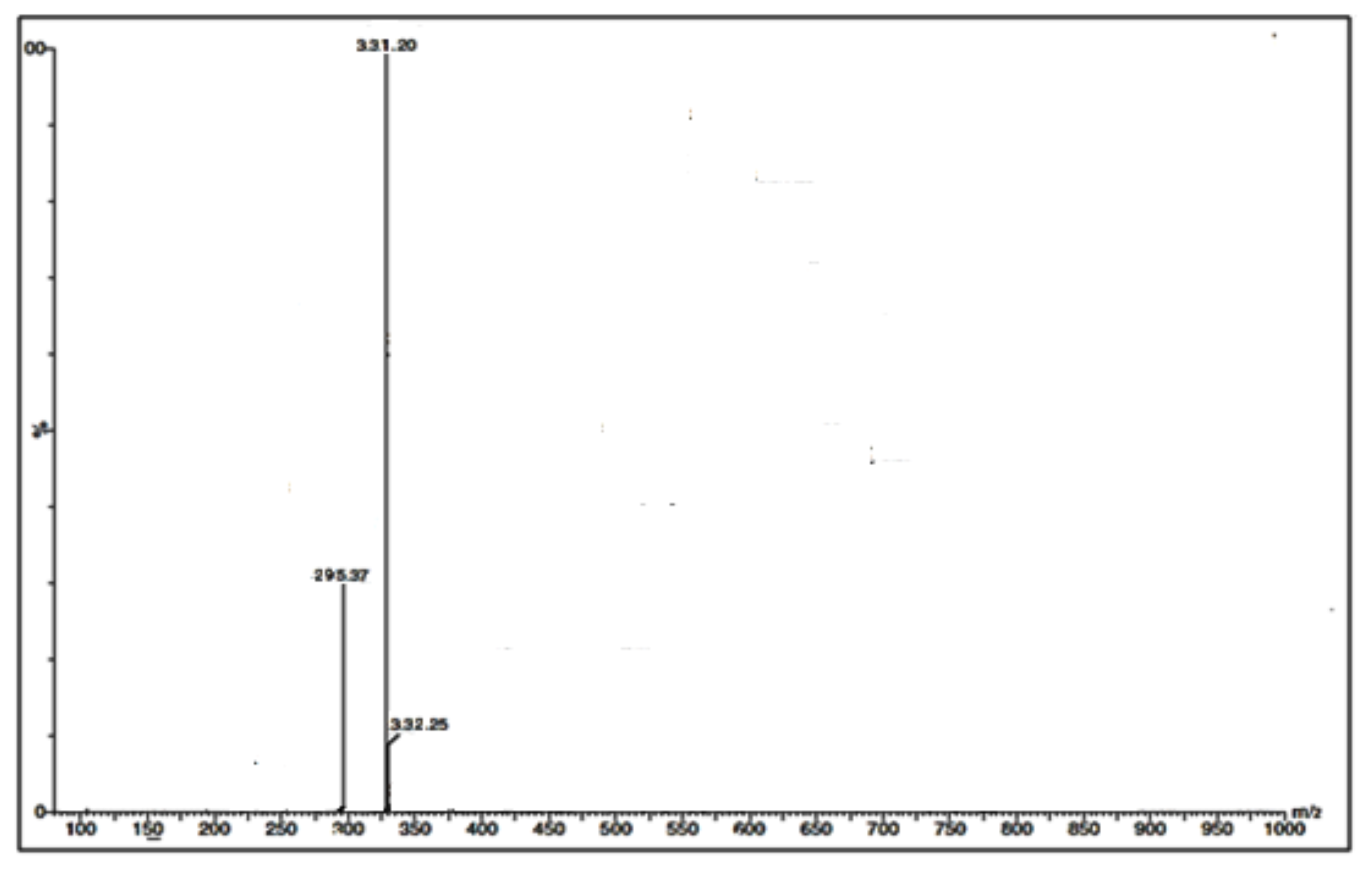

Supplement: Supplementary file 7 — Mass spectrum of compound 6b [file turkjchem-46-3-766s7.tif]

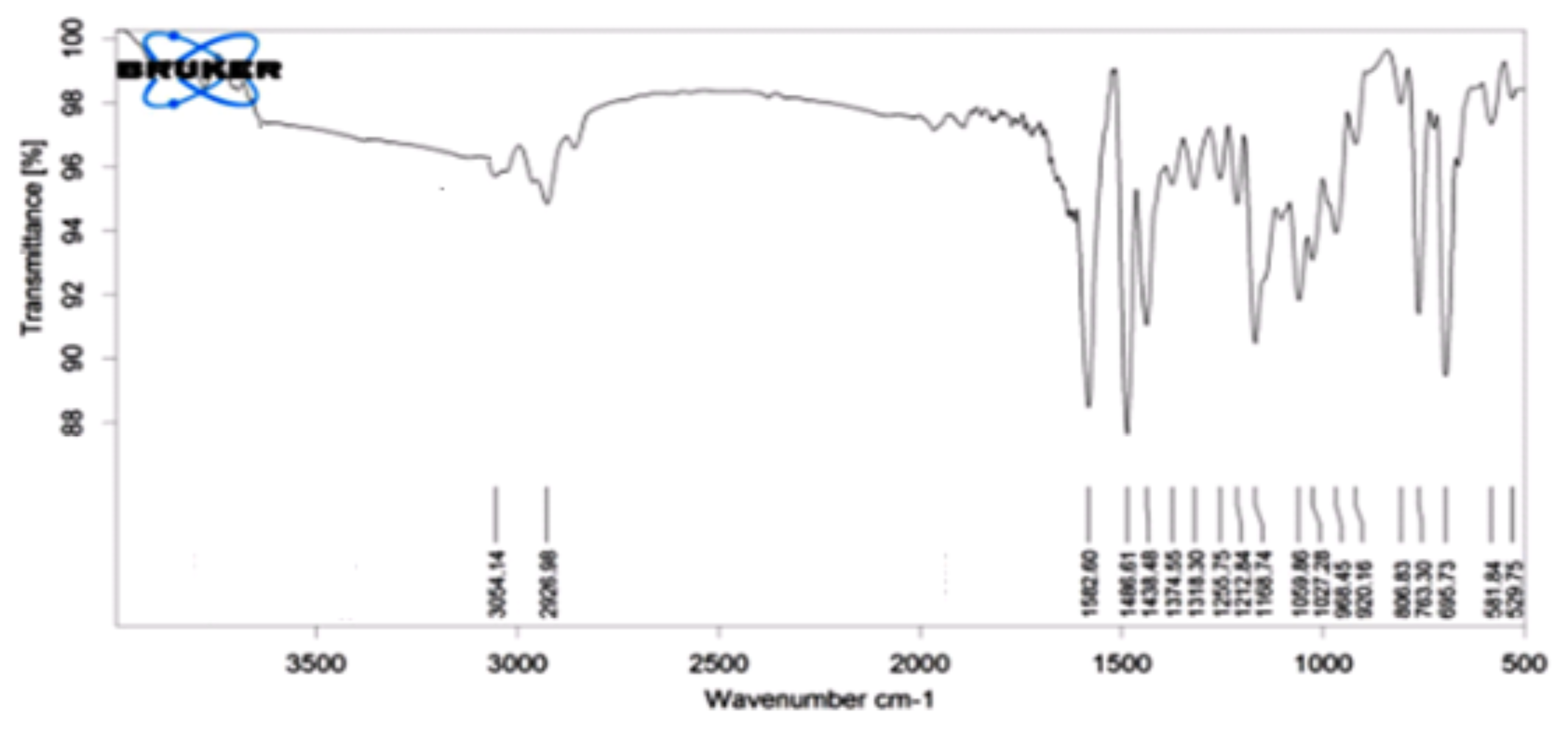

Supplement: Supplementary file 8 — IR spectrum of compound 6b [file turkjchem-46-3-766s8.tif]

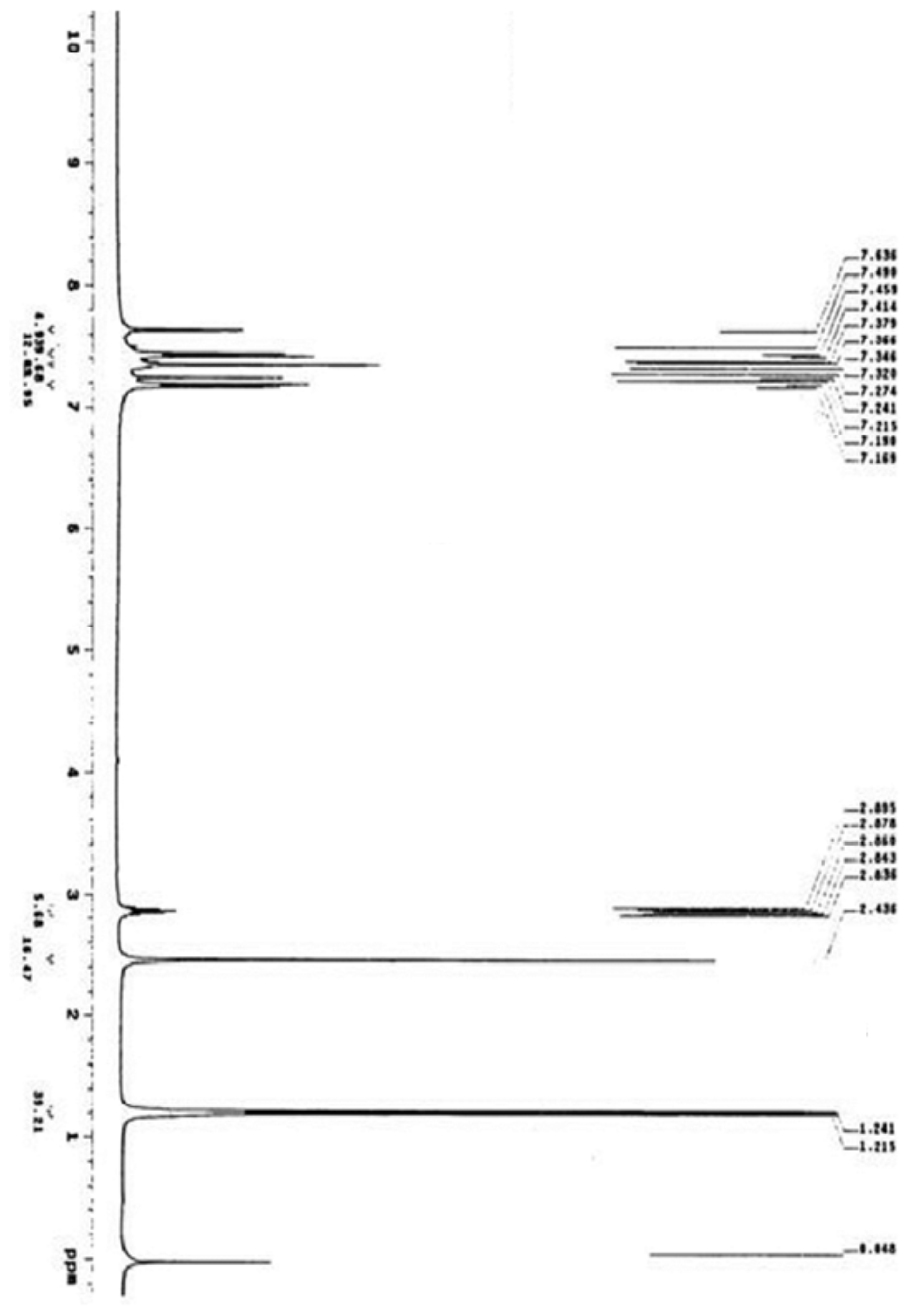

Supplement: Supplementary file 9 — 1H-NMR spectrum of compound 6c [file turkjchem-46-3-766s9.tif]

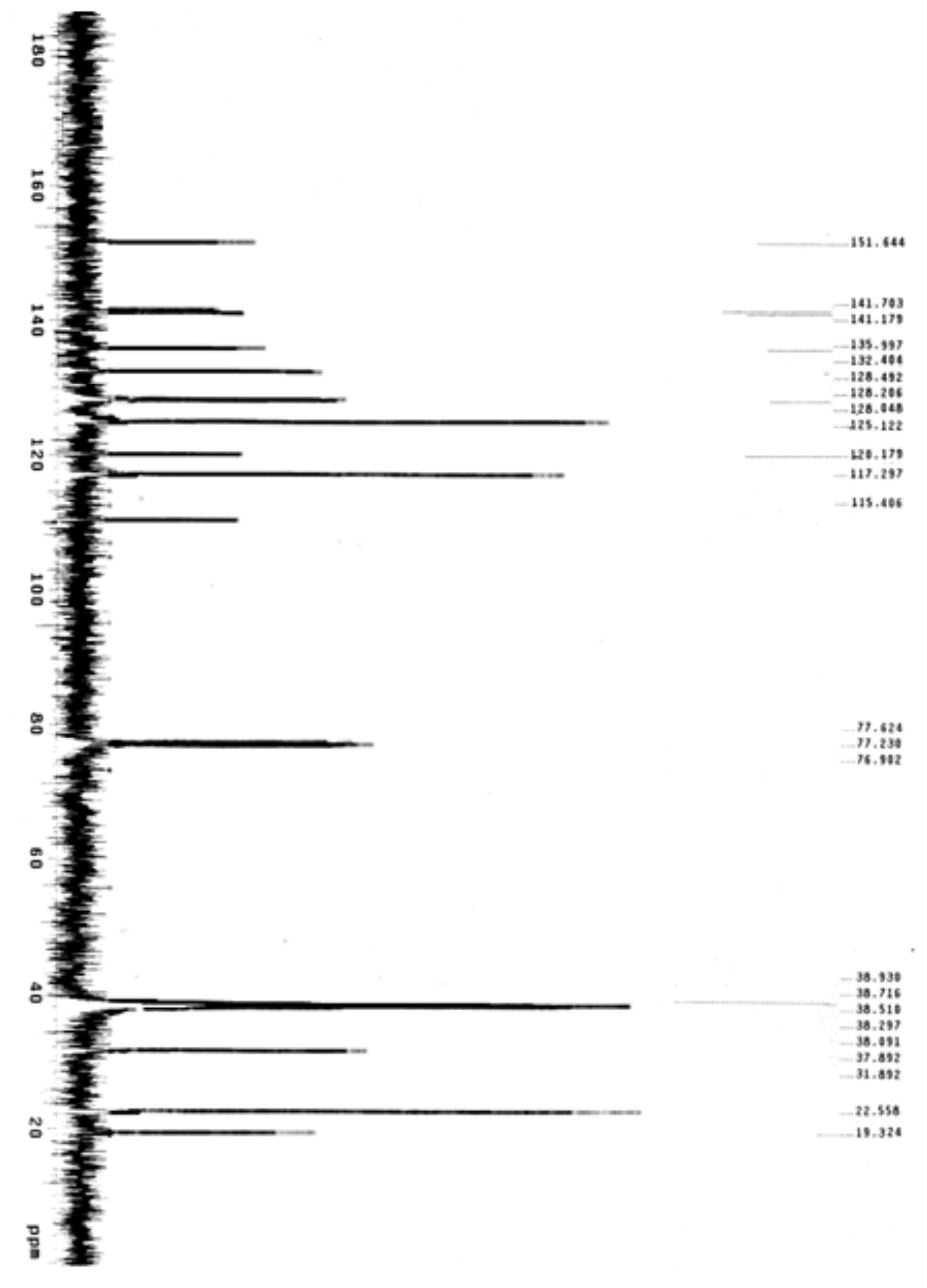

Supplement: Supplementary file 10 — 13C-NMR spectrum of compound 6c [file turkjchem-46-3-766s10.tif]

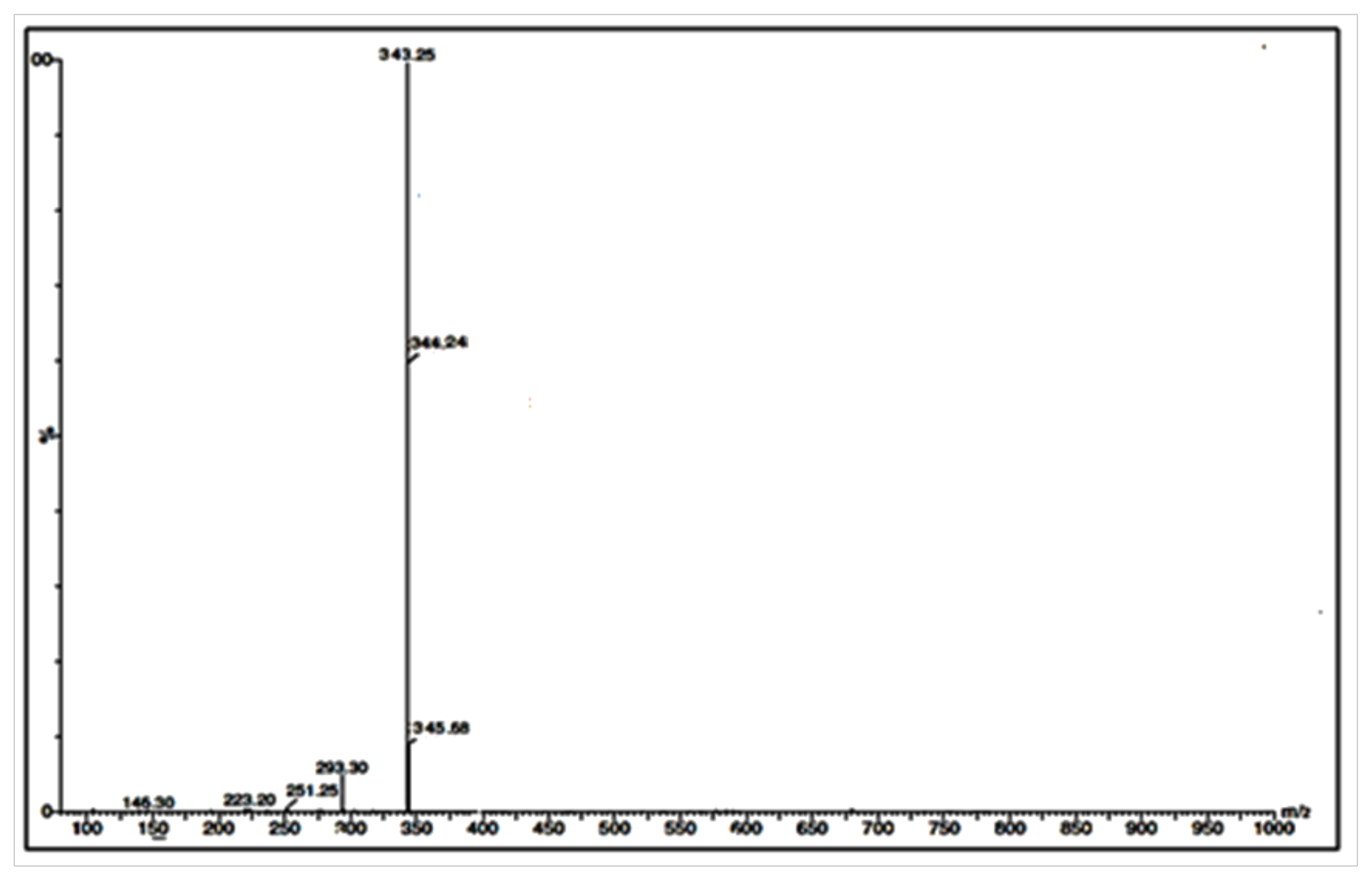

Supplement: Supplementary file 11 — Mass spectrum of compound 6c [file turkjchem-46-3-766s11.tif]

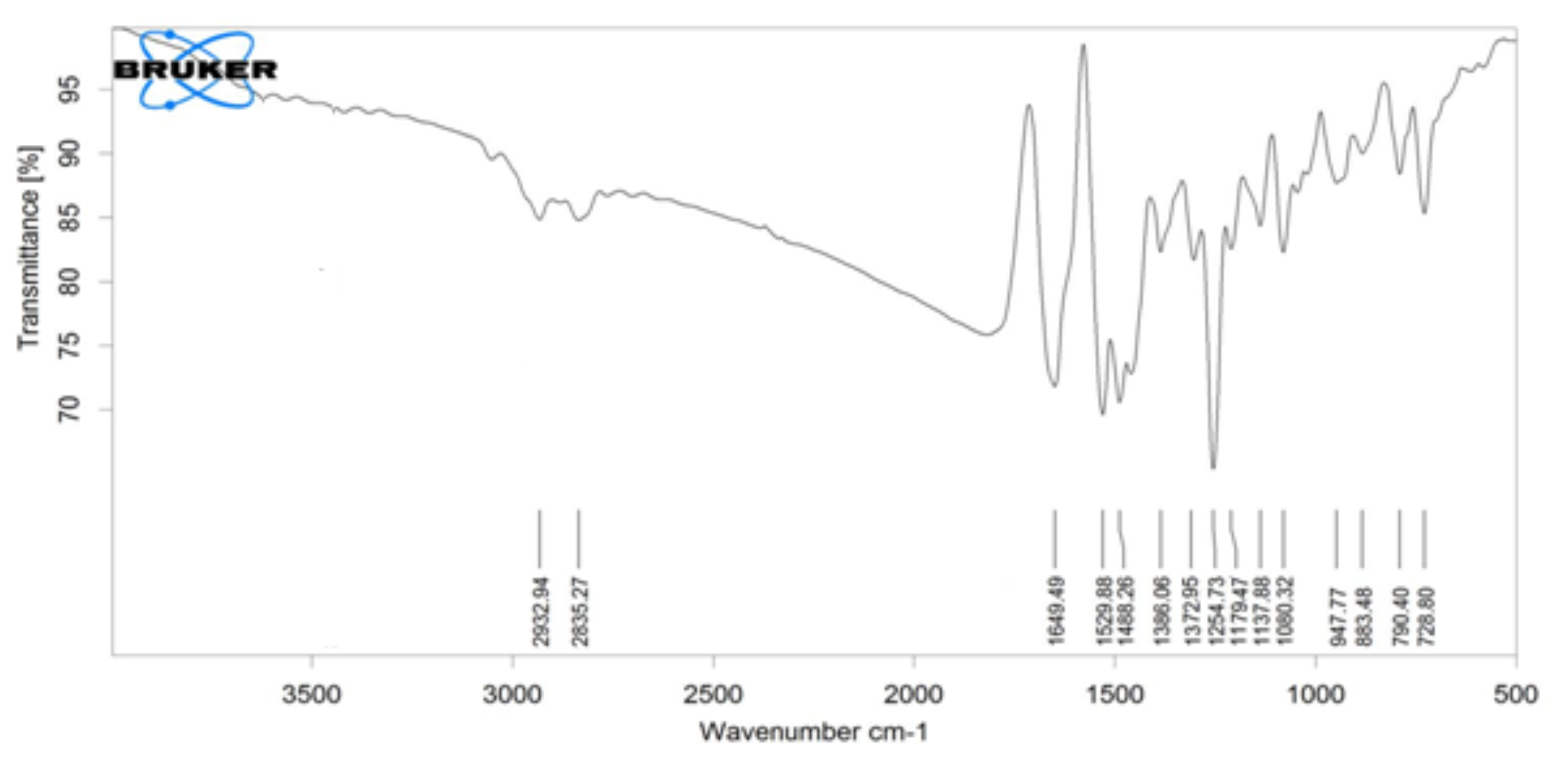

Supplement: Supplementary file 12 — IR spectrum of compound 6c [file turkjchem-46-3-766s12.tif]

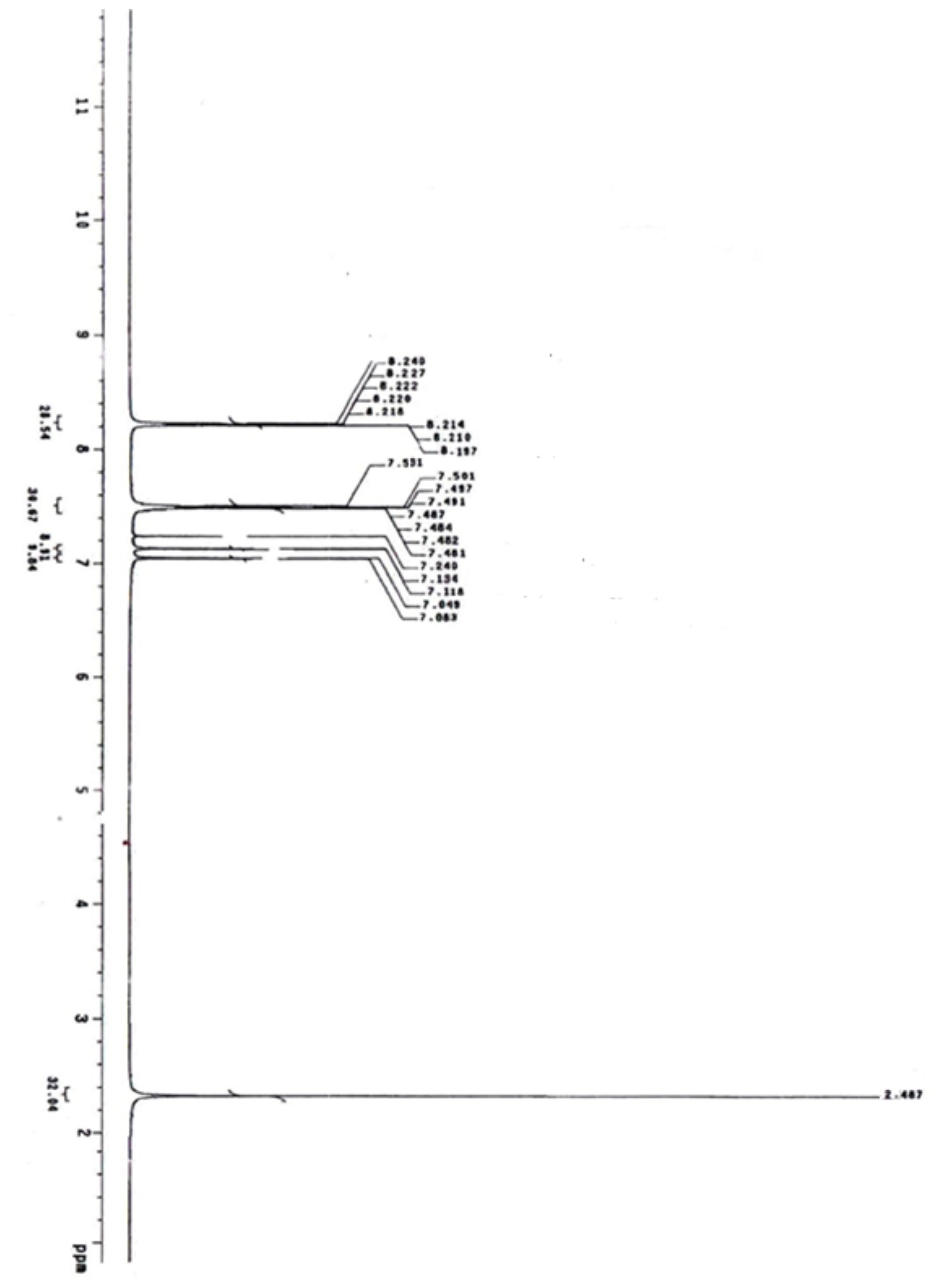

Supplement: Supplementary file 13 — 1H-NMR spectrum of compound 6d [file turkjchem-46-3-766s13.tif]

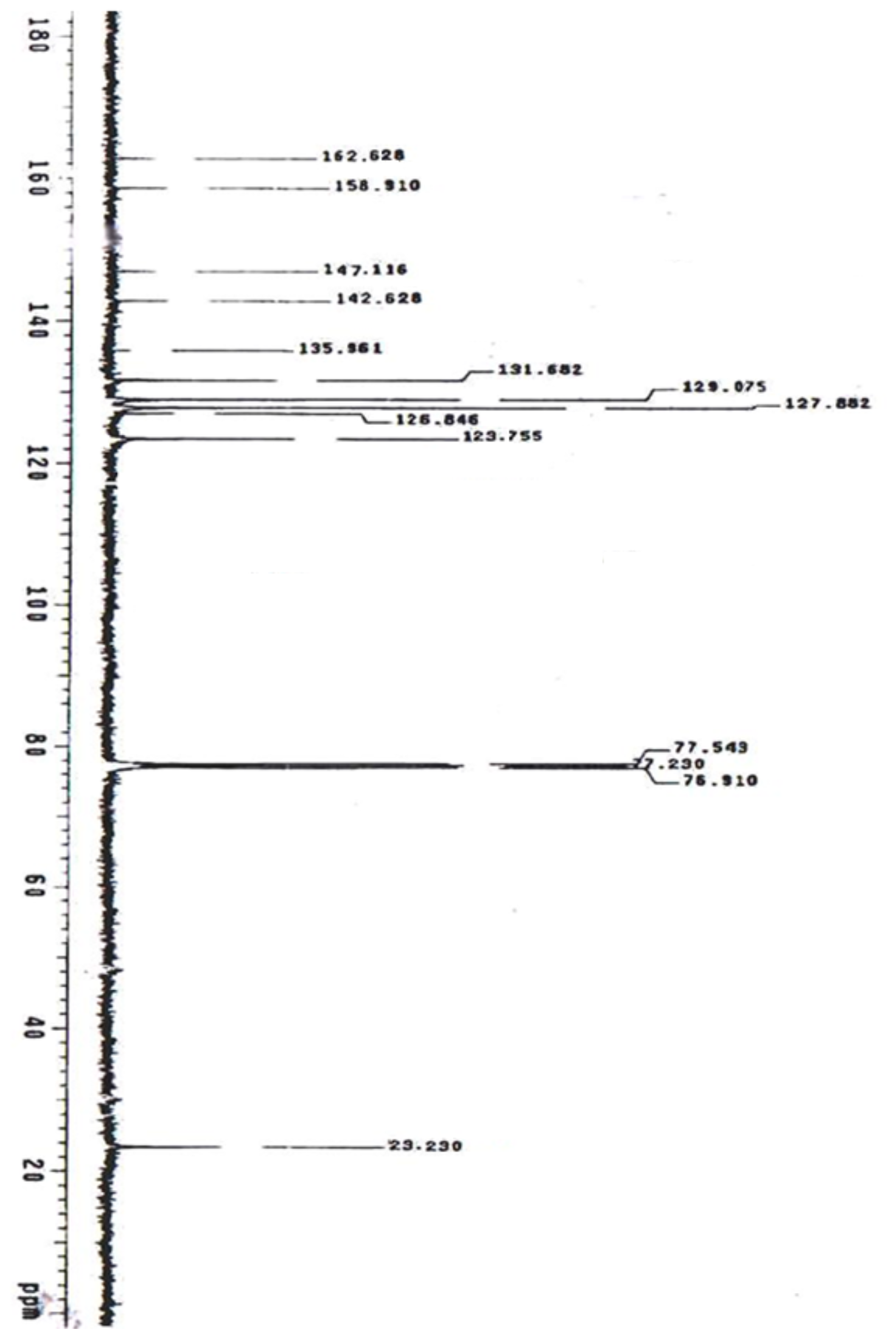

Supplement: Supplementary file 14 — 13C-NMR spectrum of compound 6d [file turkjchem-46-3-766s14.tif]

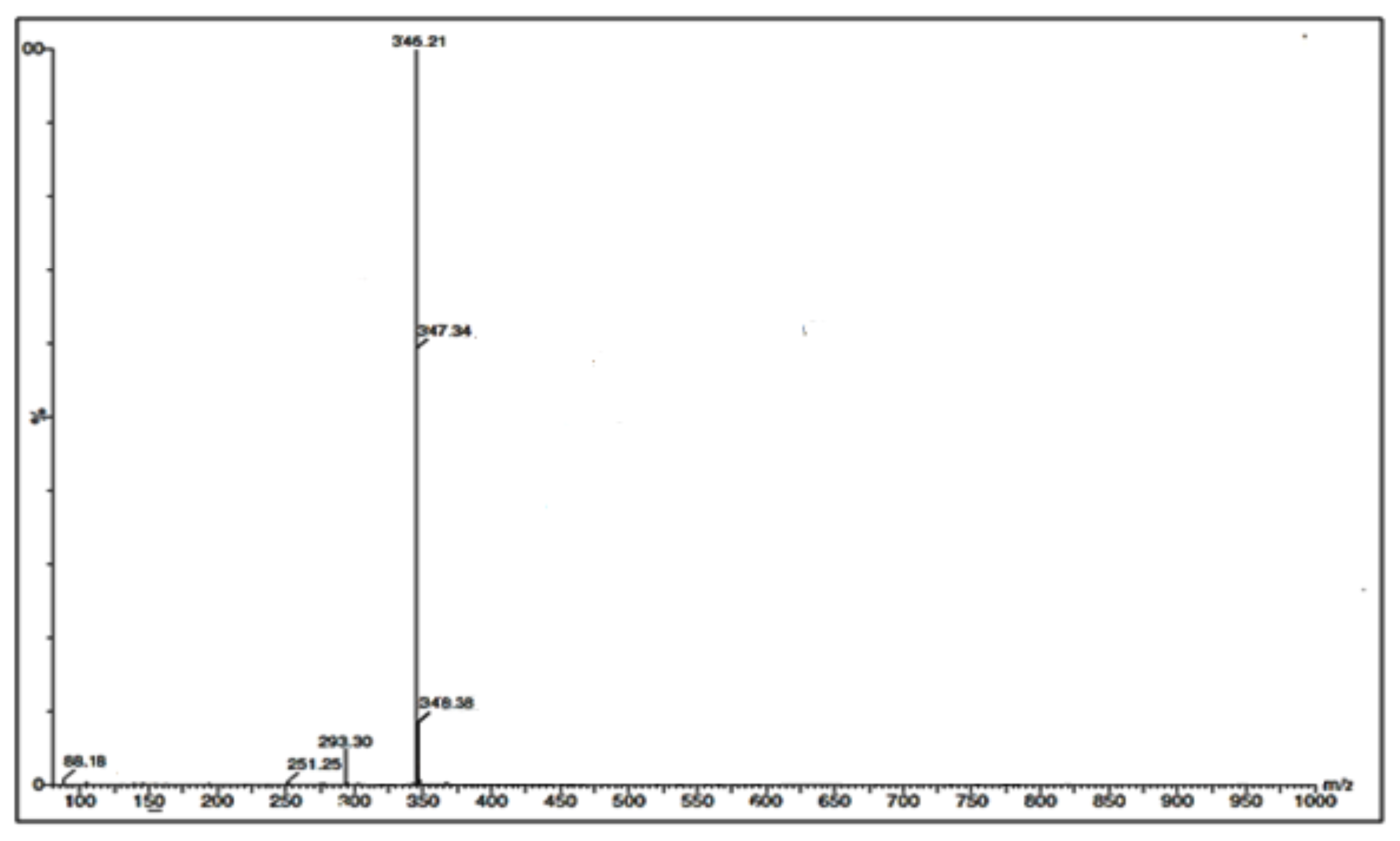

Supplement: Supplementary file 15 — Mass spectrum of compound 6d [file turkjchem-46-3-766s15.tif]

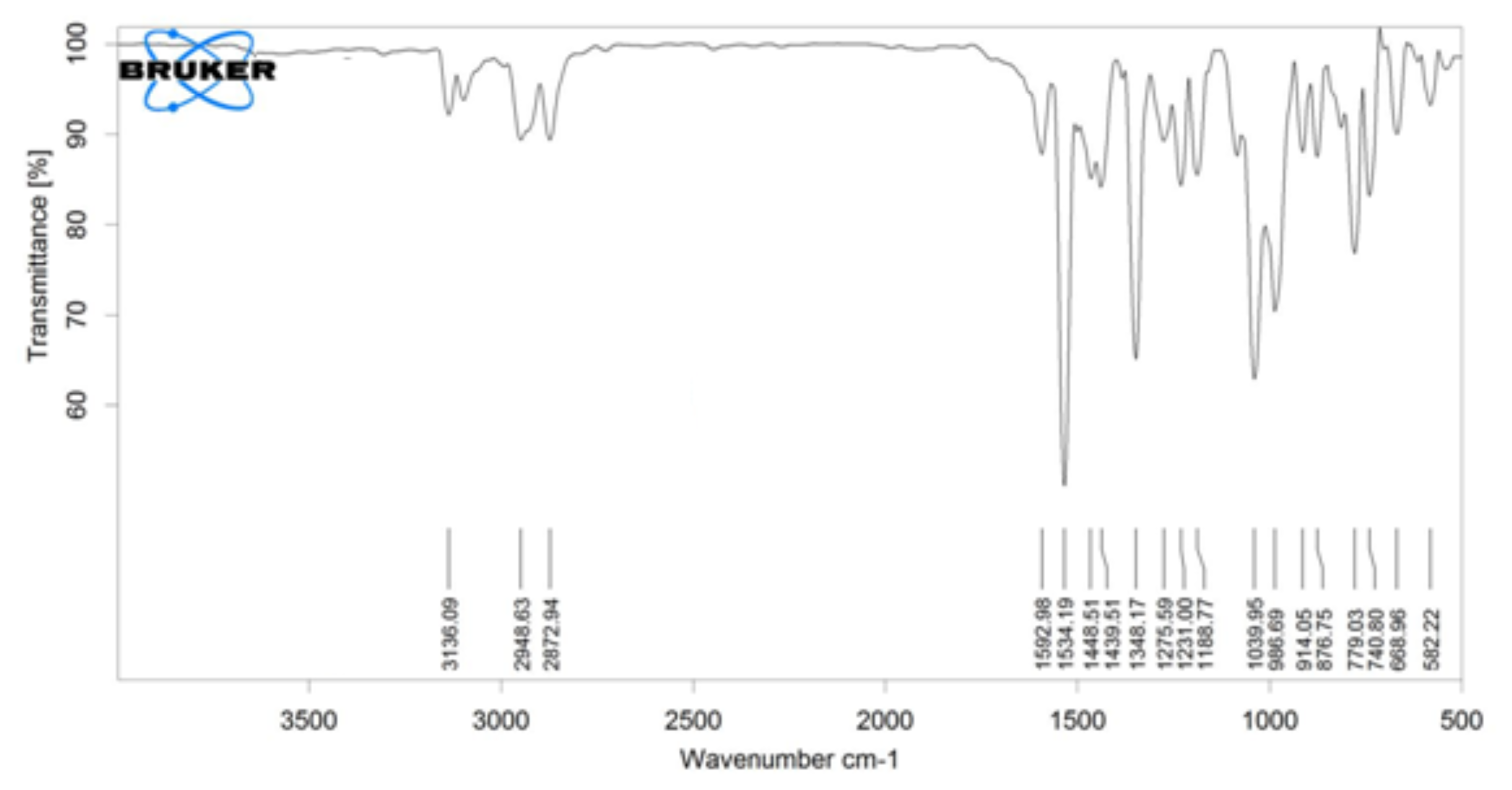

Supplement: Supplementary file 16 — IR spectrum of compound 6d [file turkjchem-46-3-766s16.tif]

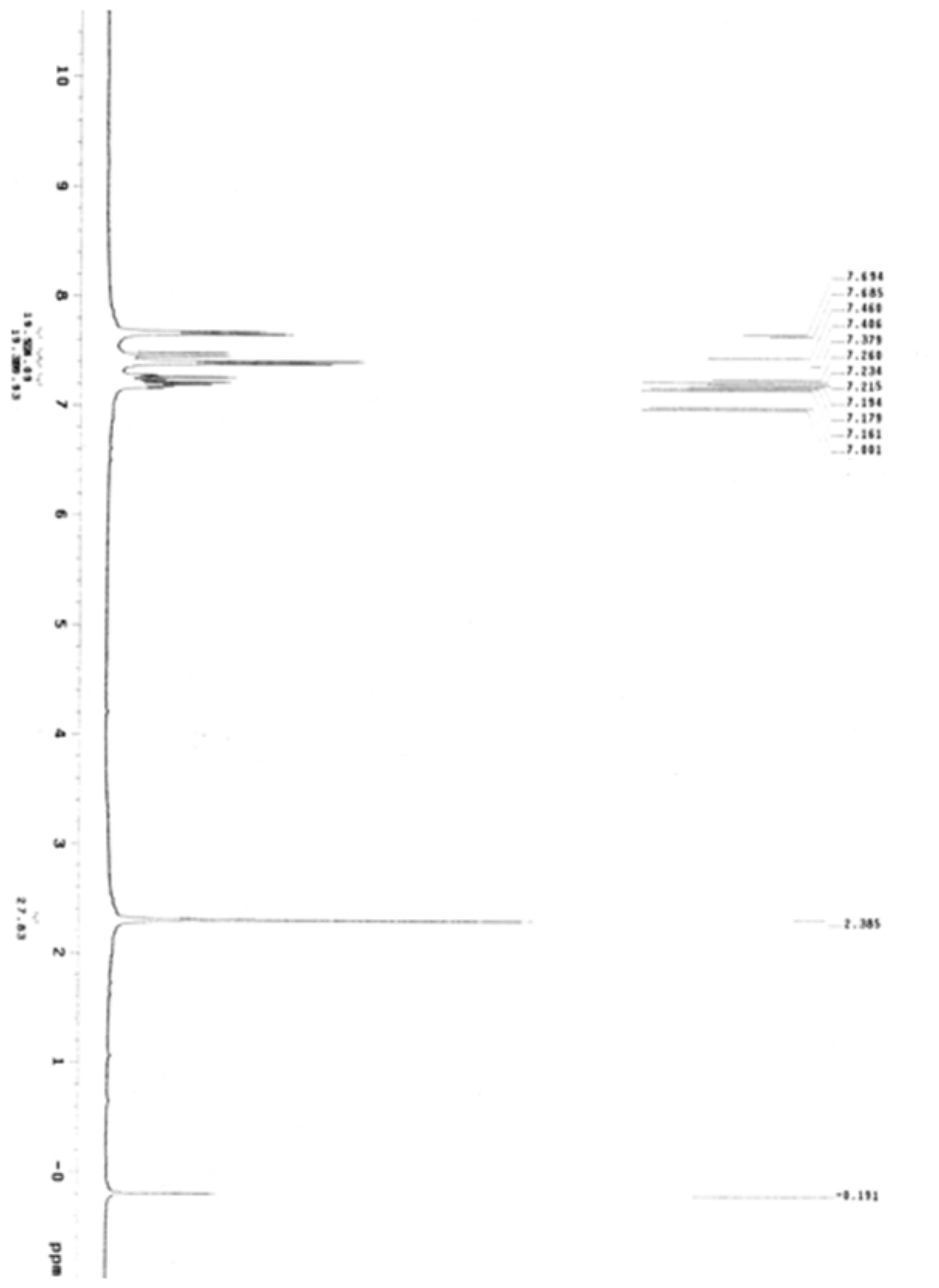

Supplement: Supplementary file 17 — 1H-NMR spectrum of compound 6e [file turkjchem-46-3-766s17.tif]

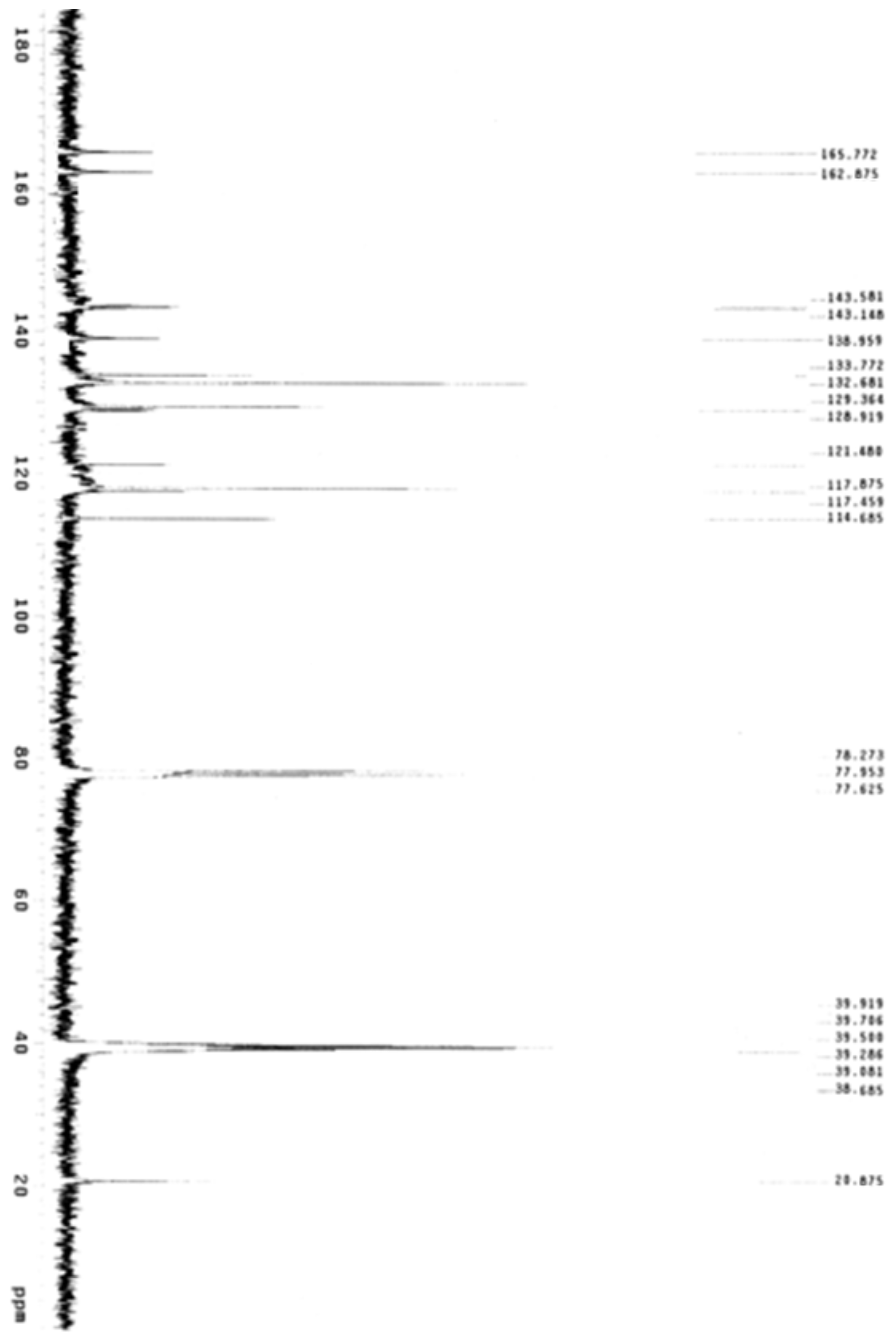

Supplement: Supplementary file 18 — 13C-NMR spectrum of compound 6e [file turkjchem-46-3-766s18.tif]

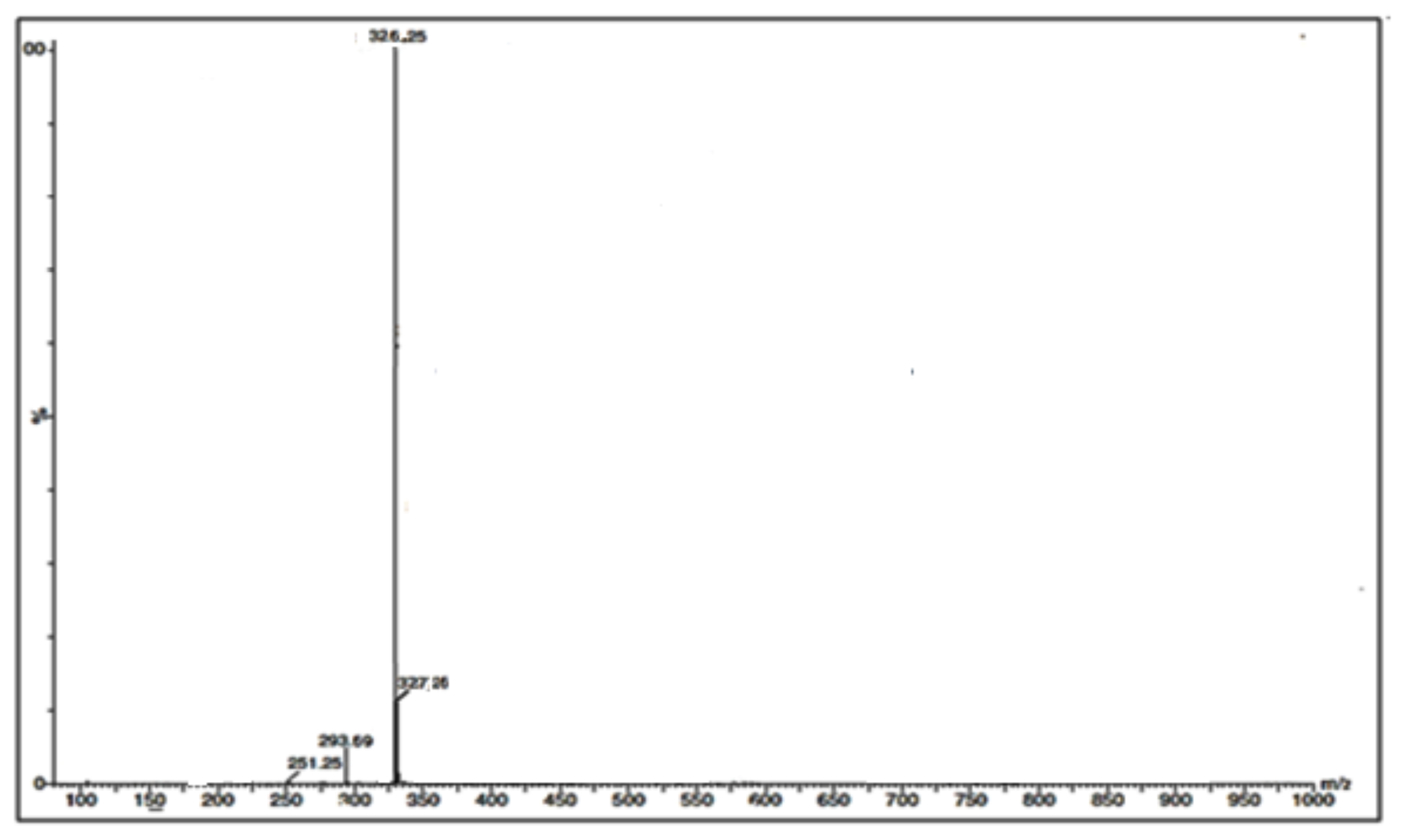

Supplement: Supplementary file 19 — Mass spectrum of compound 6e [file turkjchem-46-3-766s19.tif]

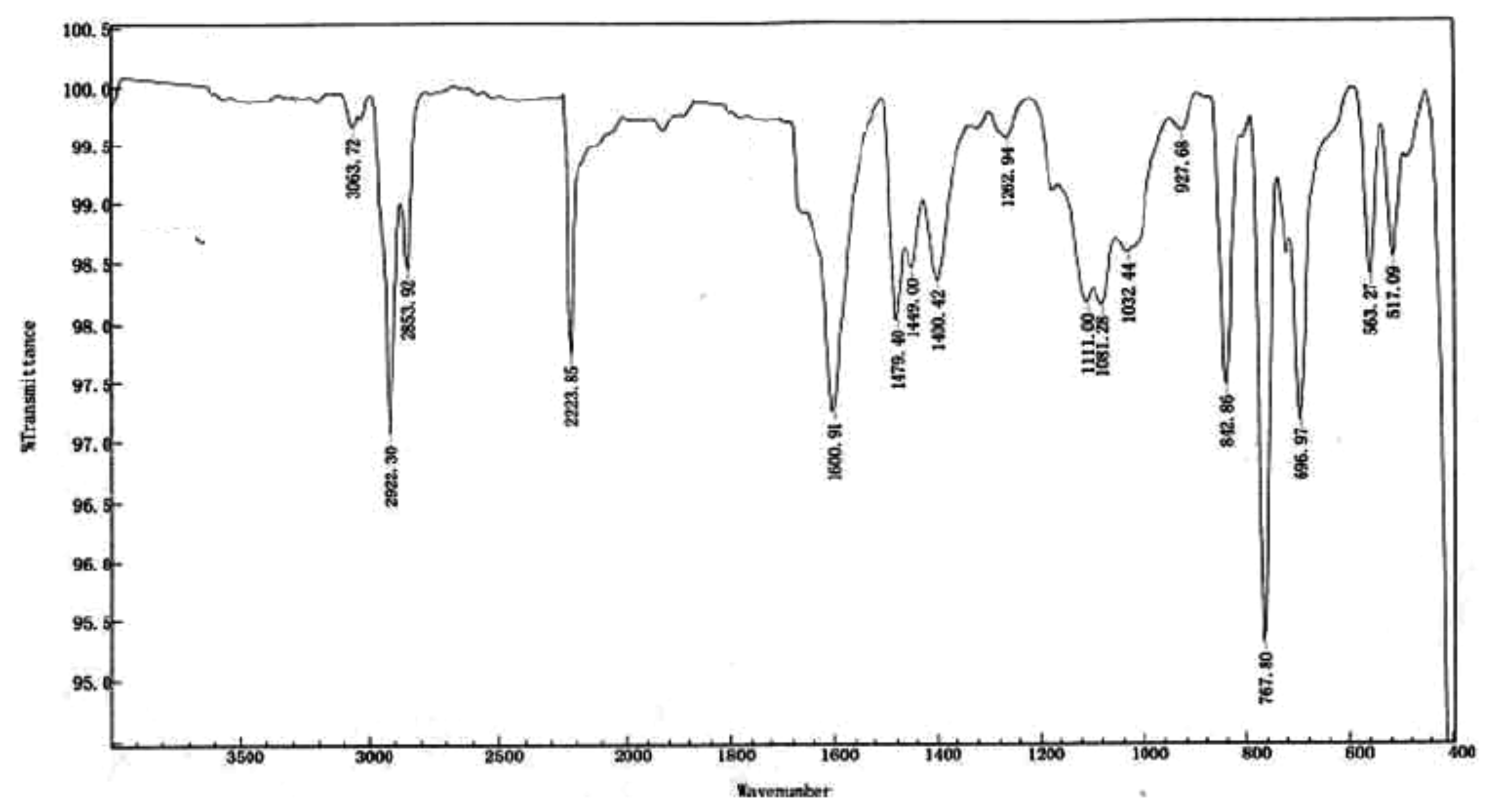

Supplement: Supplementary file 20 — IR spectrum of compound 6e [file turkjchem-46-3-766s20.tif]

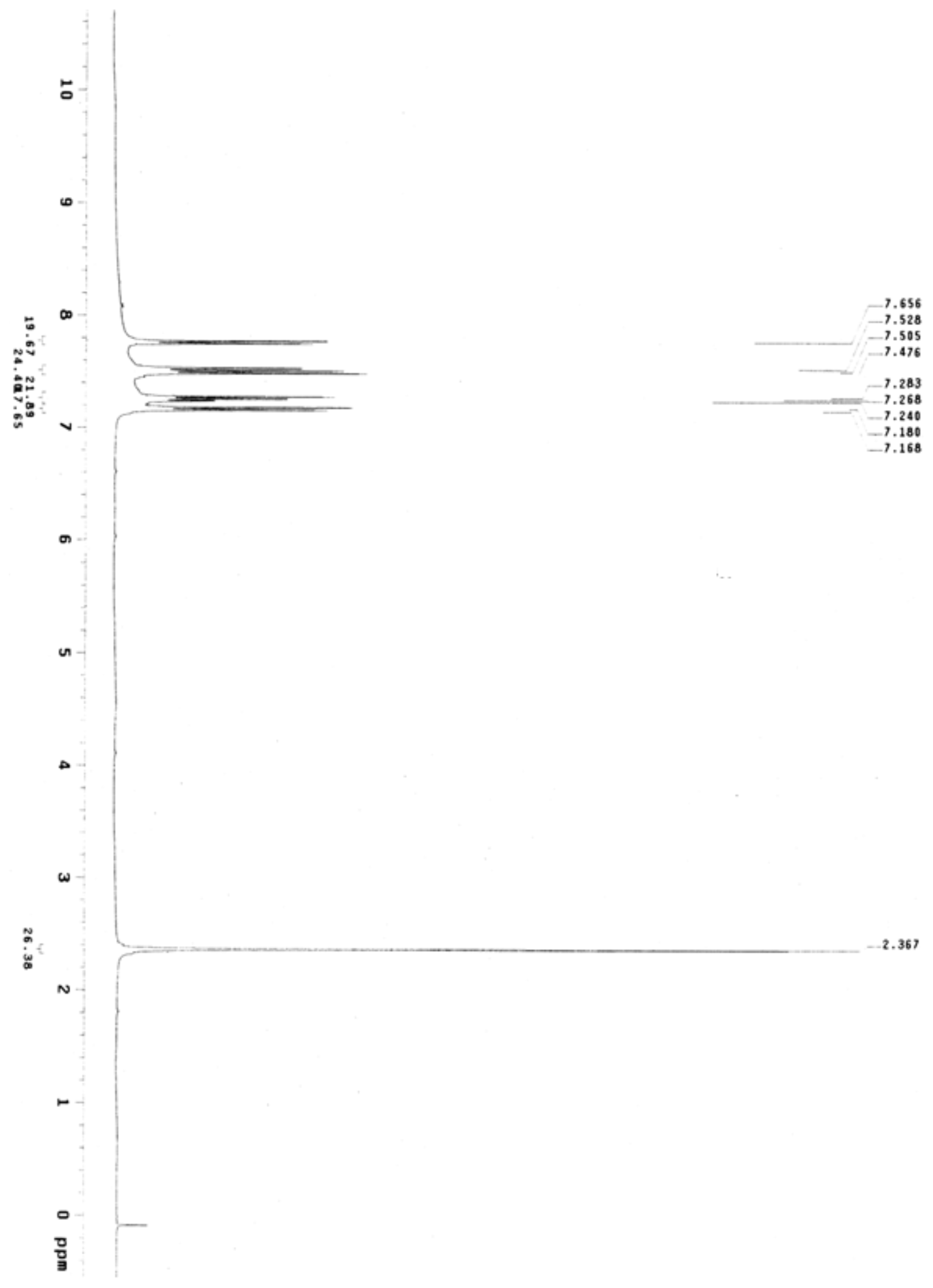

Supplement: Supplementary file 21 — 1H-NMR spectrum of compound 6f [file turkjchem-46-3-766s21.tif]

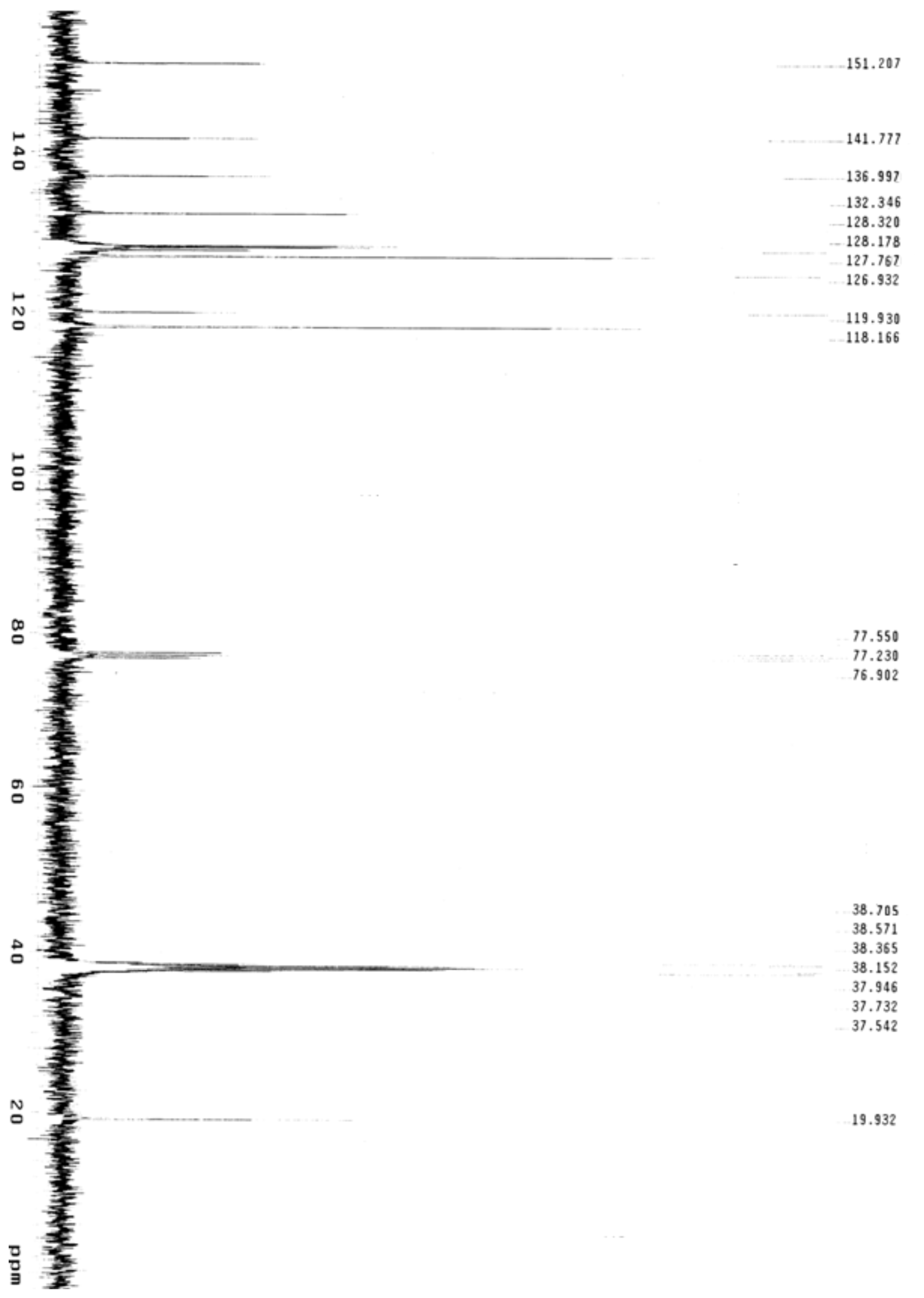

Supplement: Supplementary file 22 — 13C-NMR spectrum of compound 6f [file turkjchem-46-3-766s22.tif]

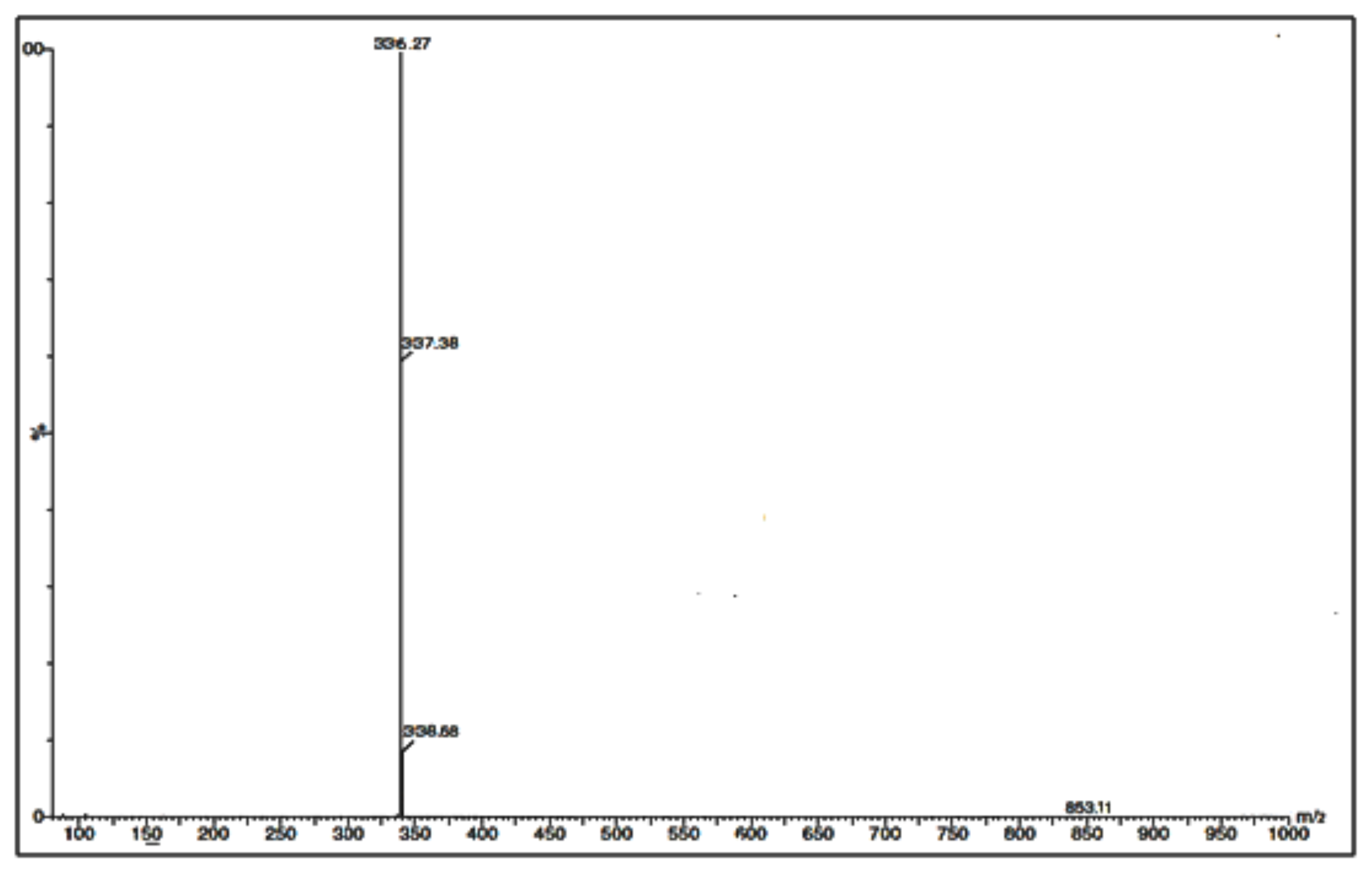

Supplement: Supplementary file 23 — Mass spectrum of compound 6f [file turkjchem-46-3-766s23.tif]

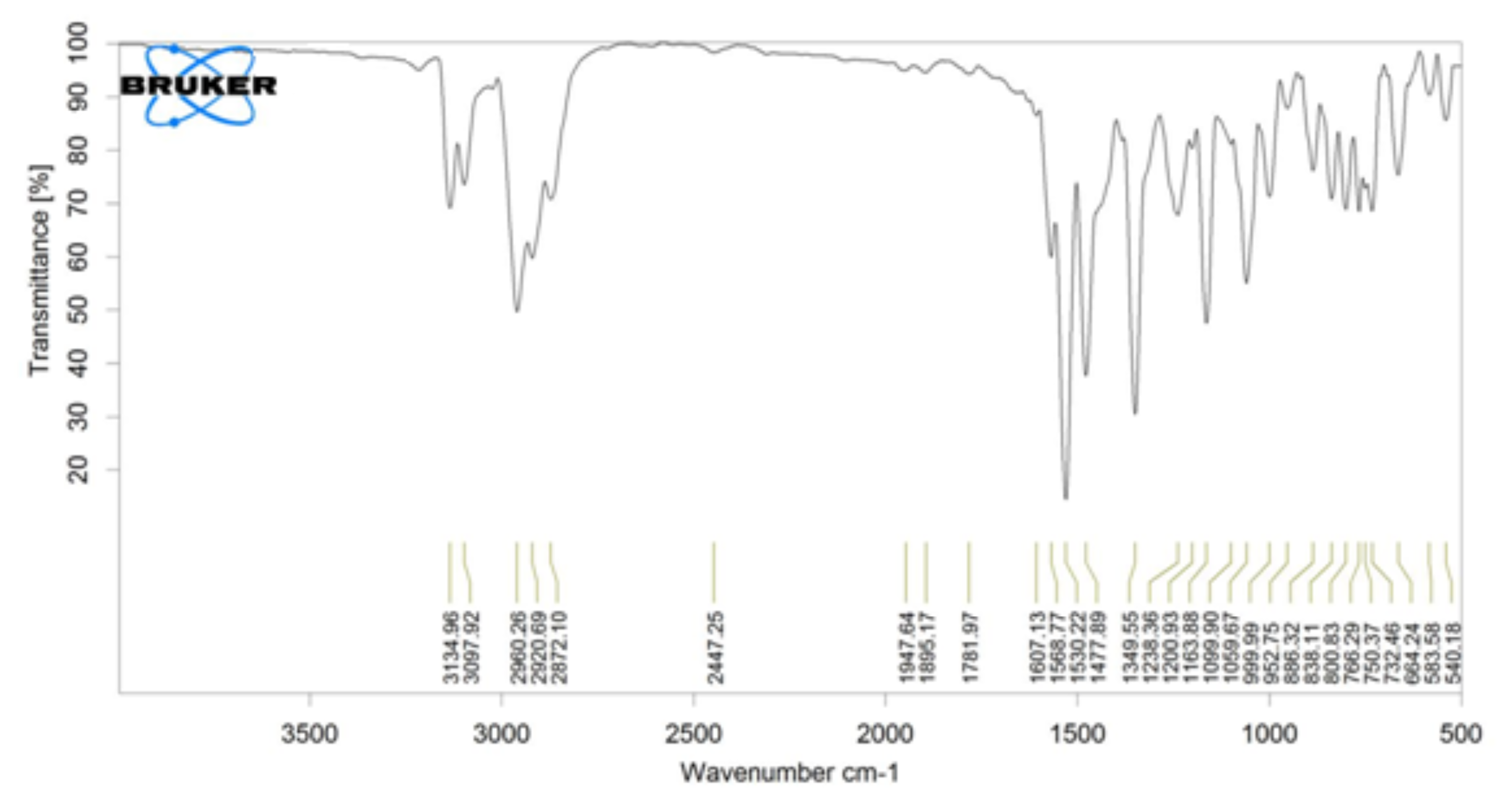

Supplement: Supplementary file 24 — IR spectrum of compound 6f [file turkjchem-46-3-766s24.tif]

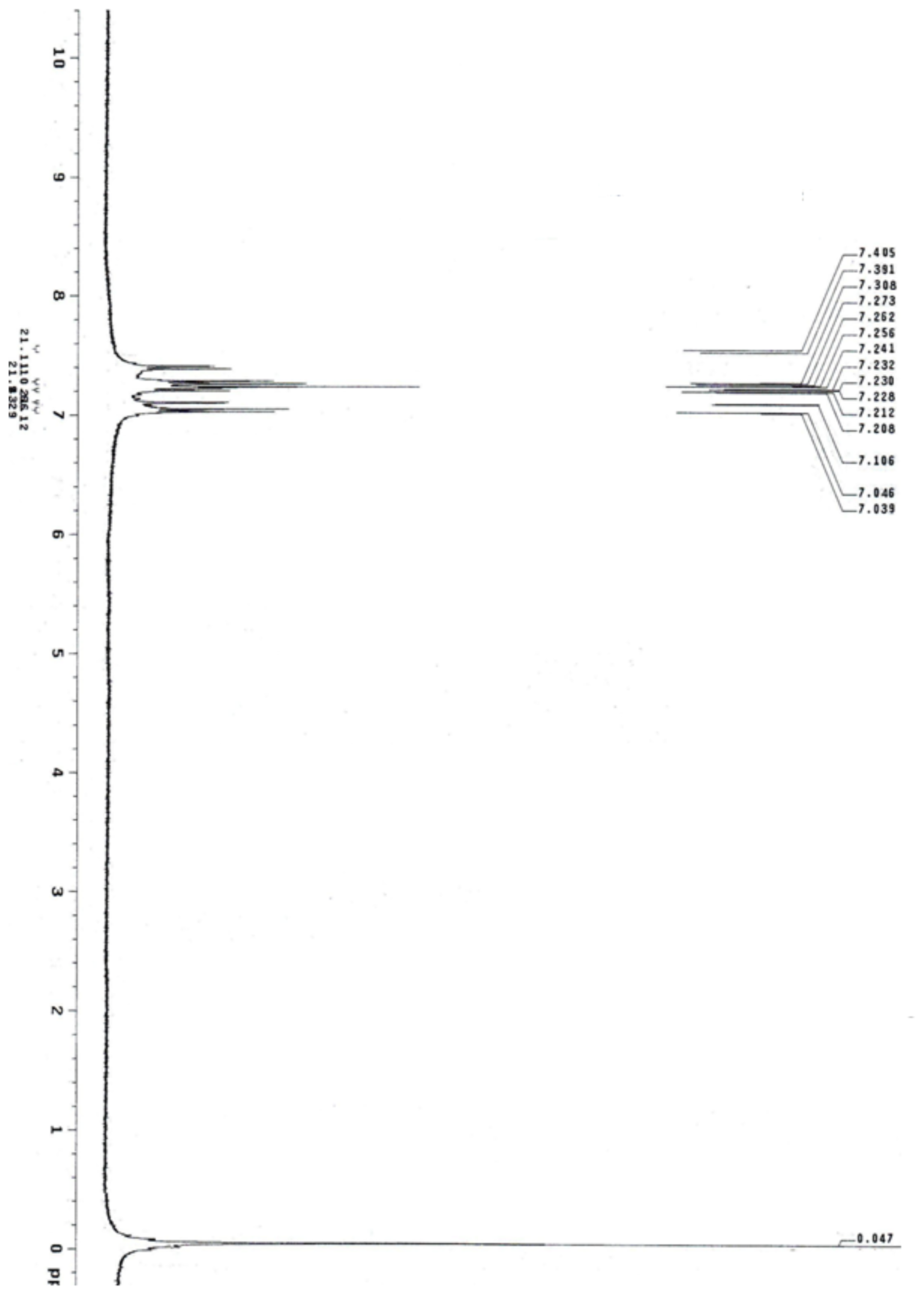

Supplement: Supplementary file 25 — 1H-NMR spectrum of compound 6g [file turkjchem-46-3-766s25.tif]

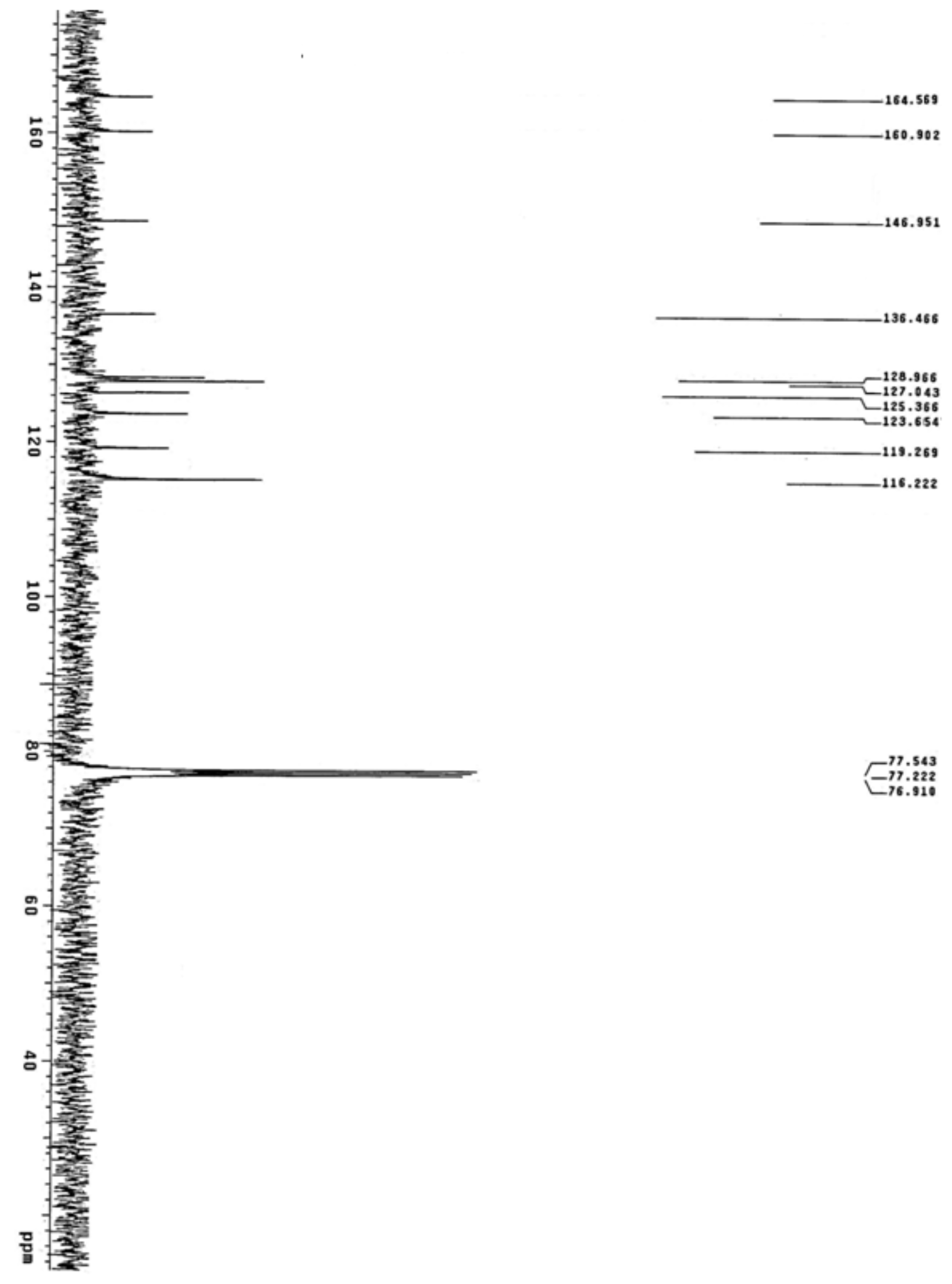

Supplement: Supplementary file 26 — 13C-NMR spectrum of compound 6g [file turkjchem-46-3-766s26.tif]

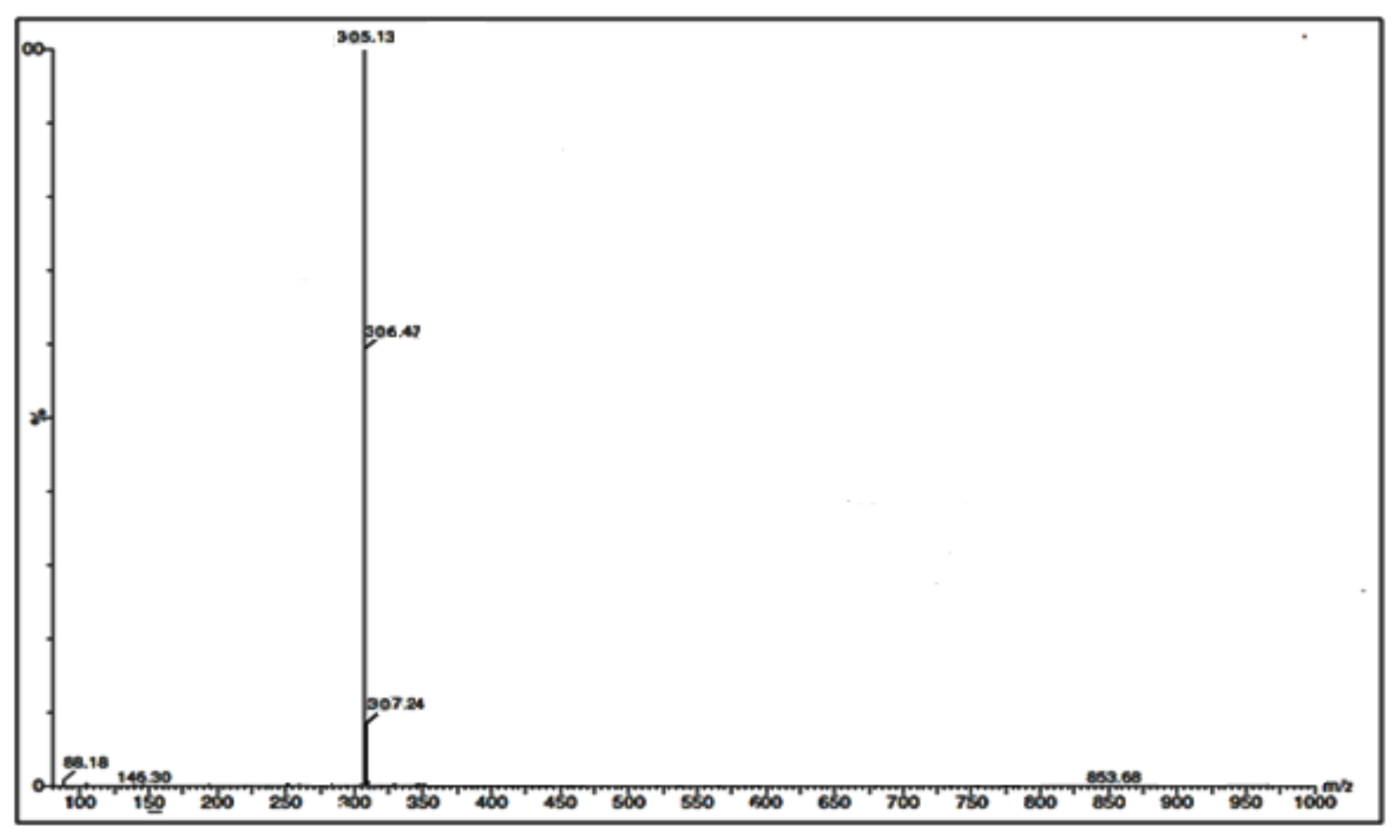

Supplement: Supplementary file 27 — Mass spectrum of compound 6g [file turkjchem-46-3-766s27.tif]

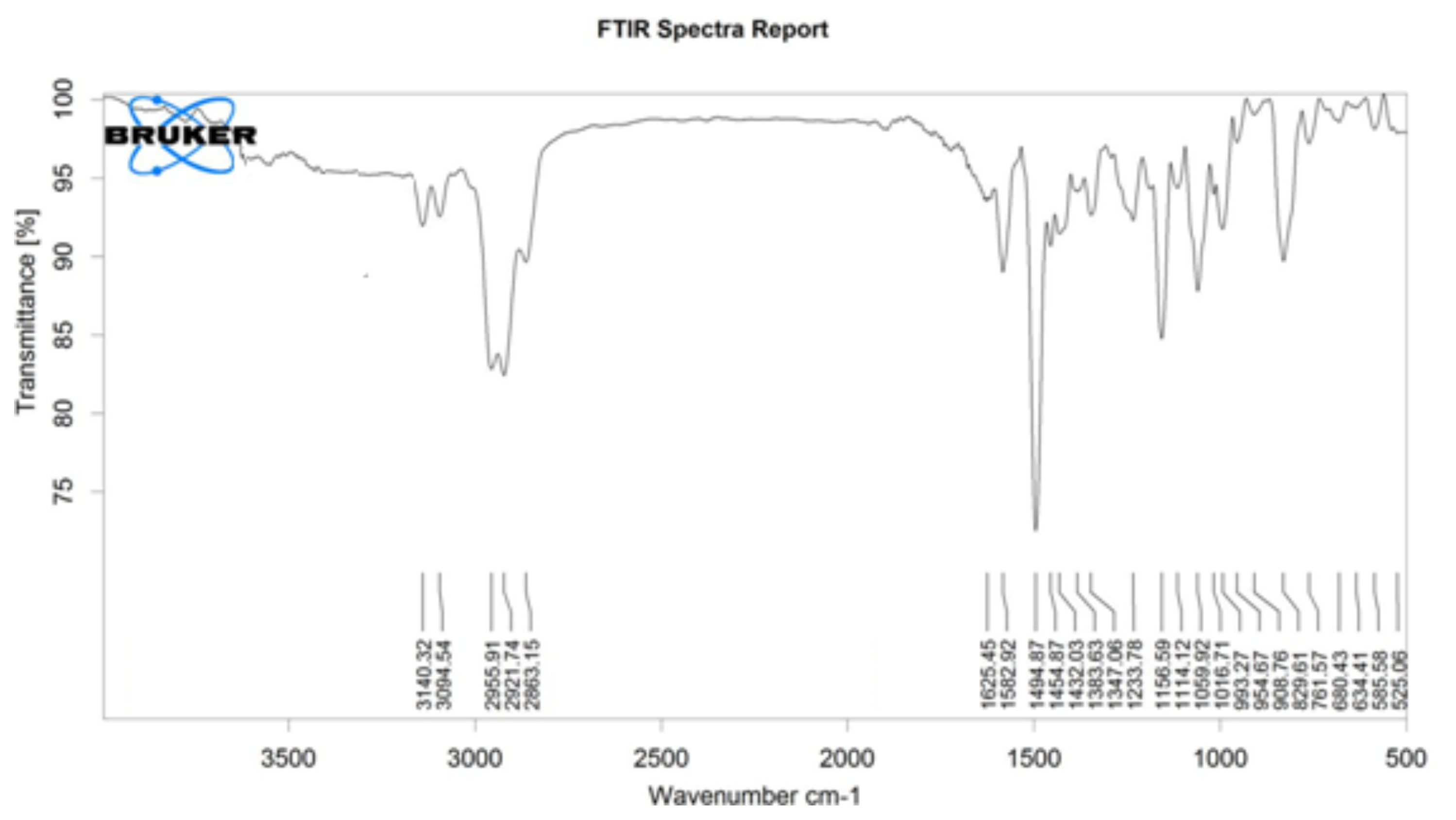

Supplement: Supplementary file 28 — IR spectrum of compound 6g [file turkjchem-46-3-766s28.tif]

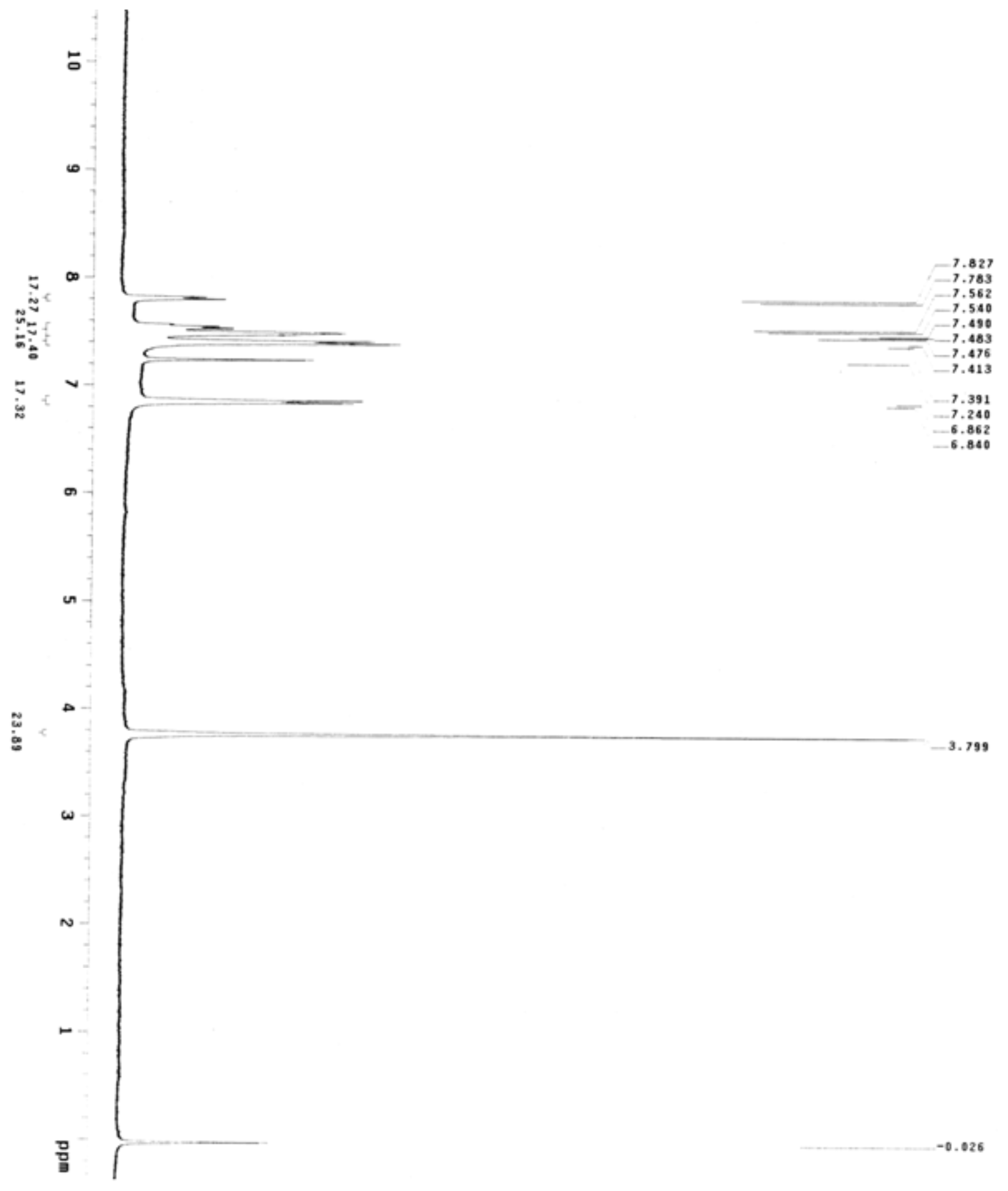

Supplement: Supplementary file 29 — 1H-NMR spectrum of compound 6h [file turkjchem-46-3-766s29.tif]

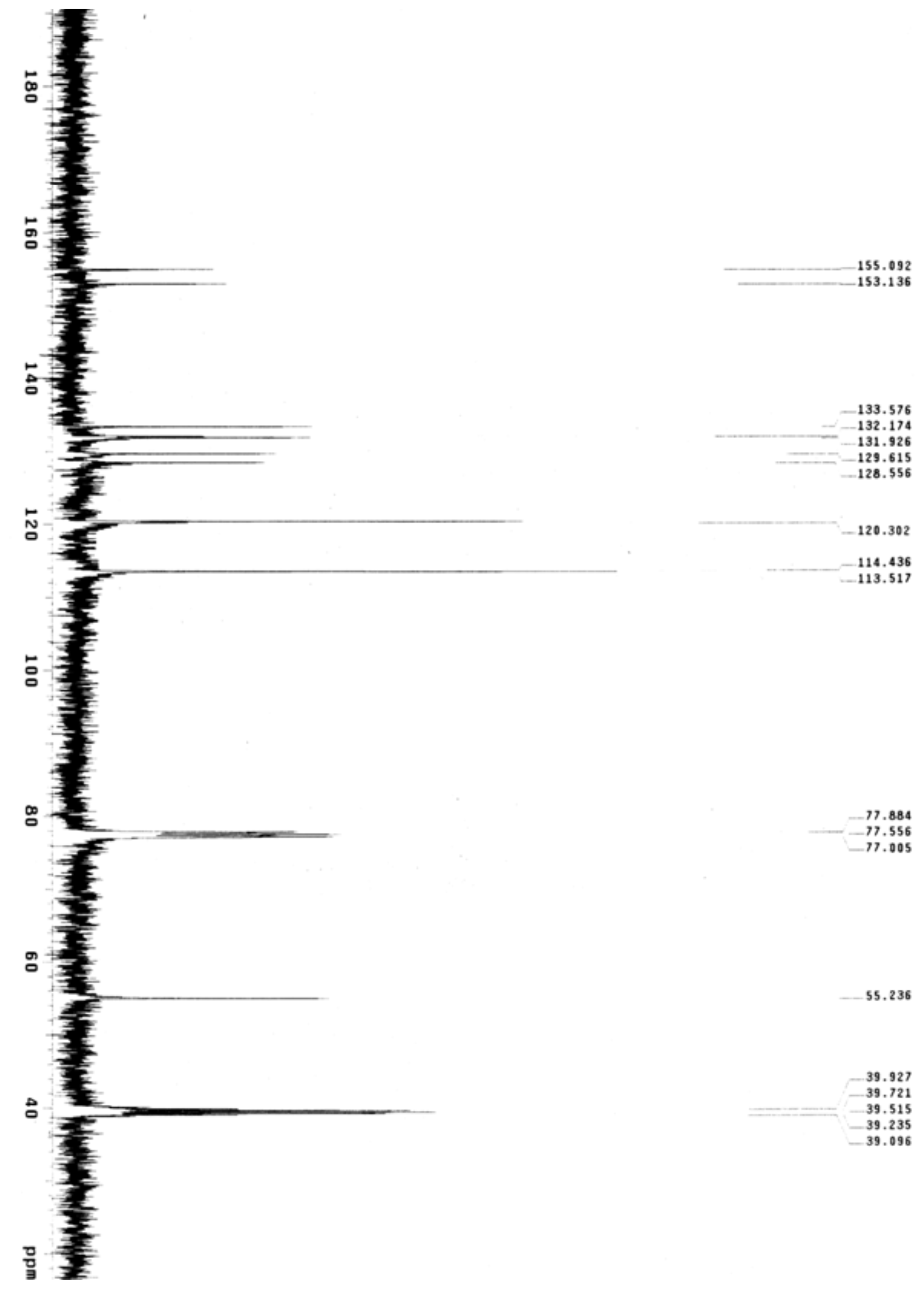

Supplement: Supplementary file 30 — 13C-NMR spectrum of compound 6h [file turkjchem-46-3-766s30.tif]

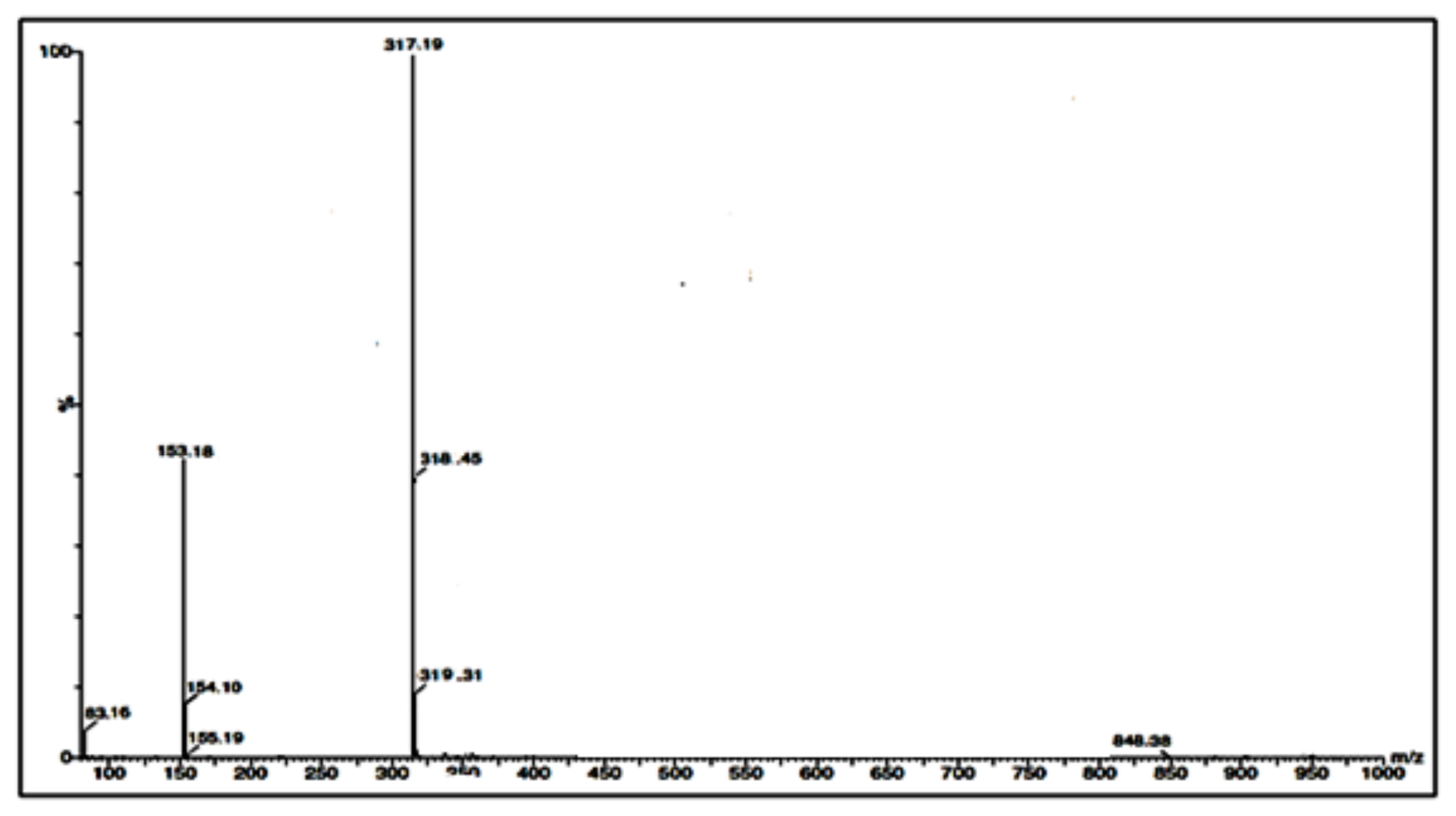

Supplement: Supplementary file 31 — Mass spectrum of compound 6h [file turkjchem-46-3-766s31.tif]

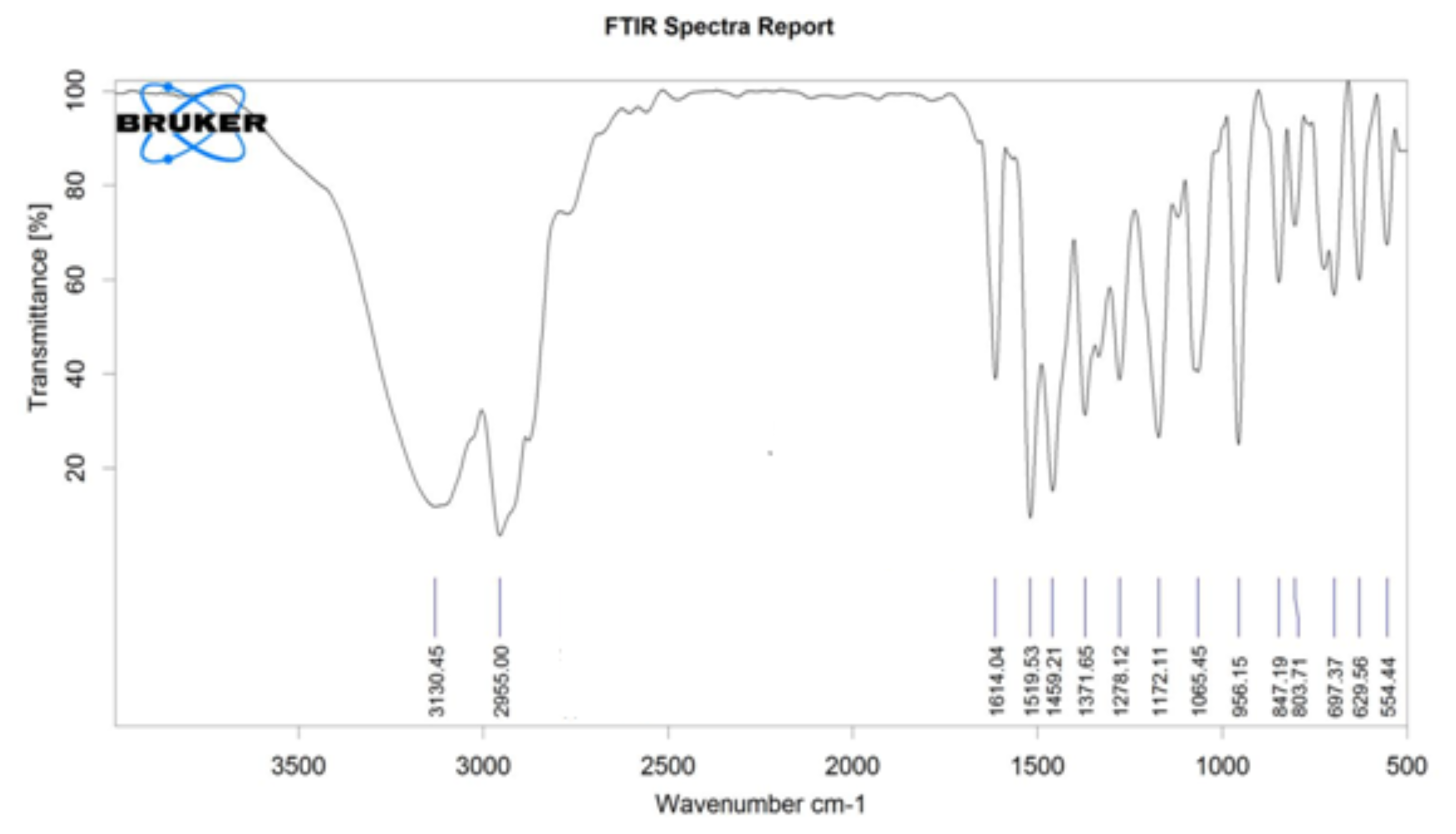

Supplement: Supplementary file 32 — IR spectrum of compound 6h [file turkjchem-46-3-766s32.tif]

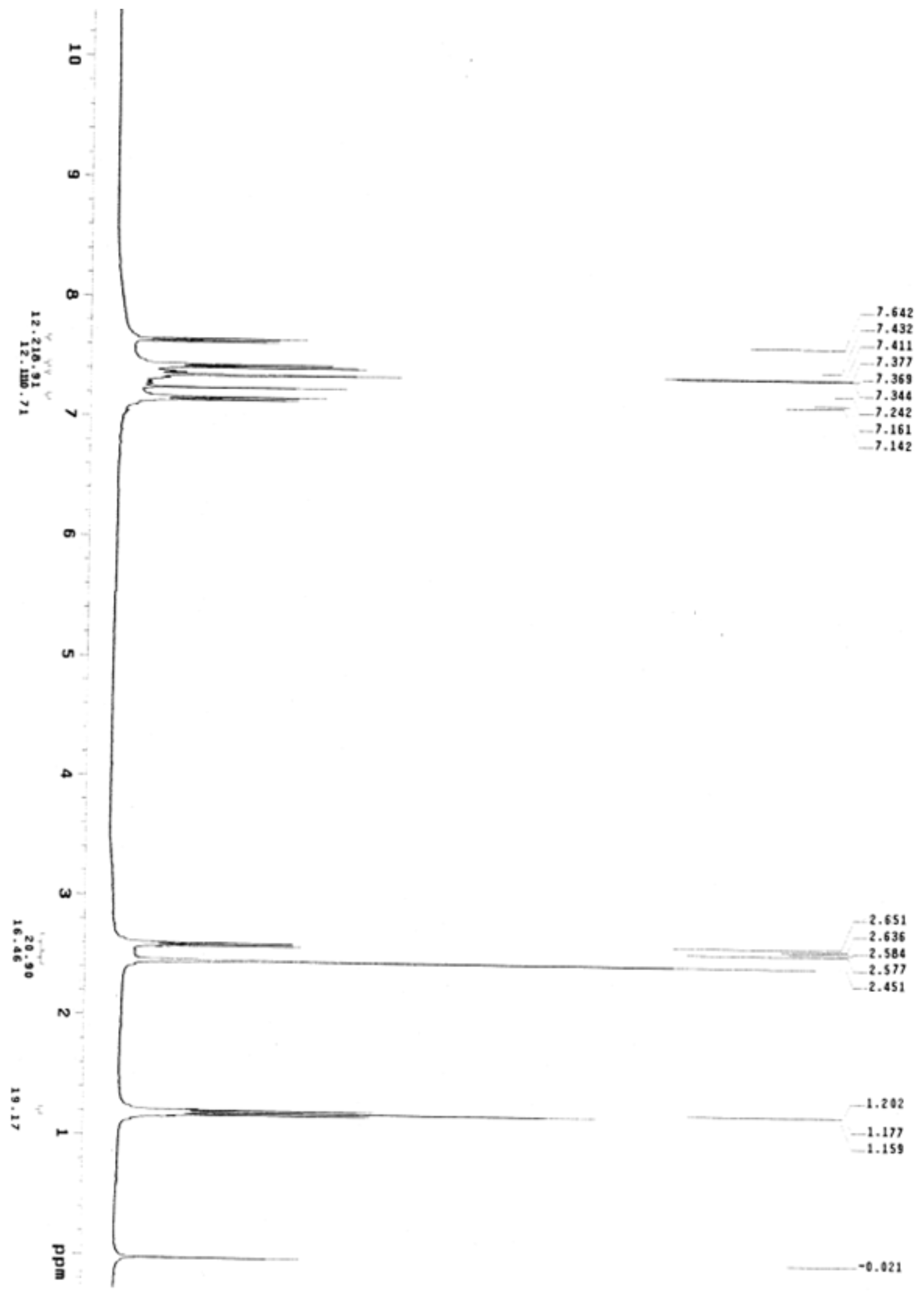

Supplement: Supplementary file 33 — 1H-NMR spectrum of compound 6i [file turkjchem-46-3-766s33.tif]

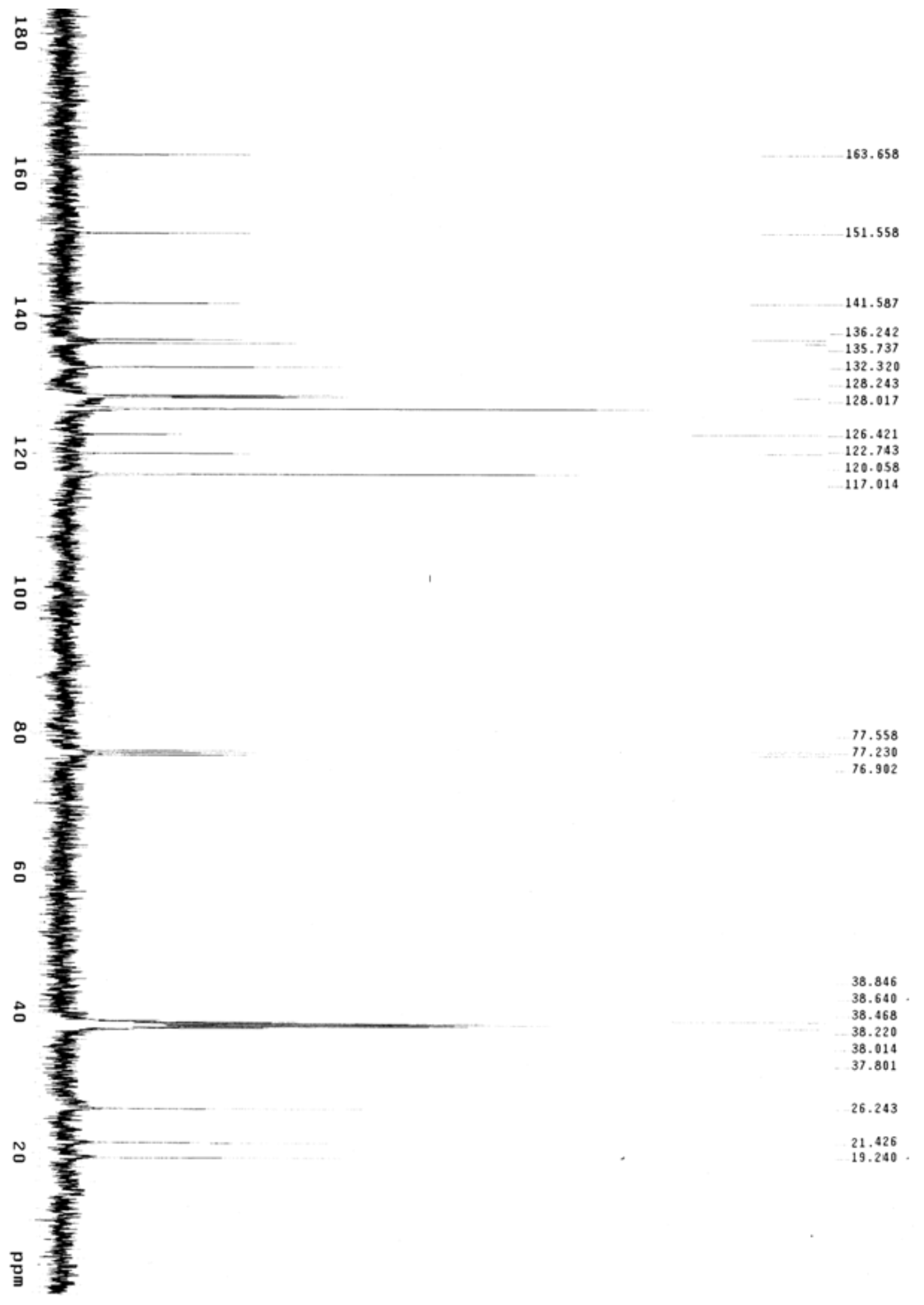

Supplement: Supplementary file 34 — 13C-NMR spectrum of compound 6i [file turkjchem-46-3-766s34.tif]

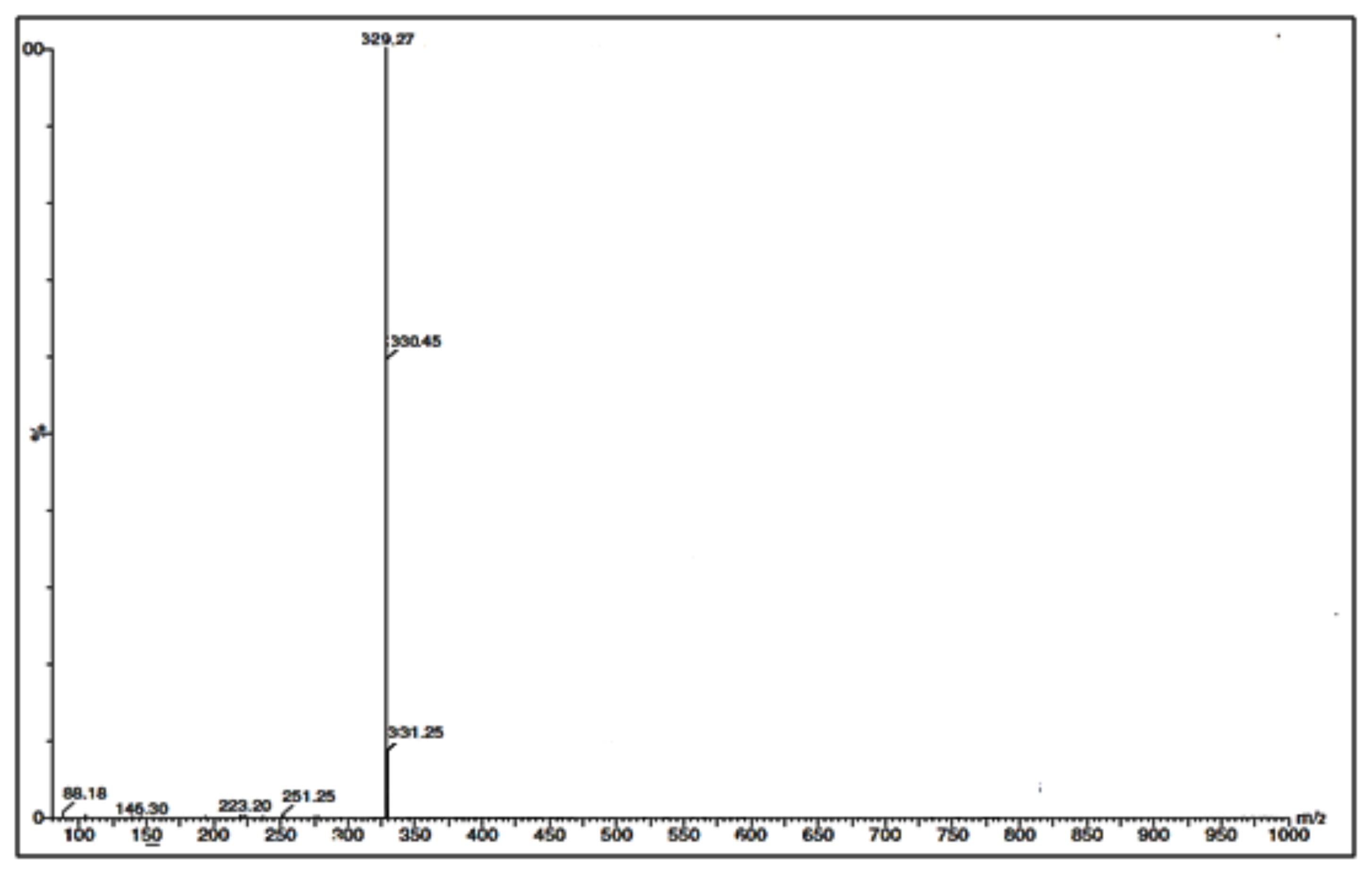

Supplement: Supplementary file 35 — Mass spectrum of compound 6i [file turkjchem-46-3-766s35.tif]

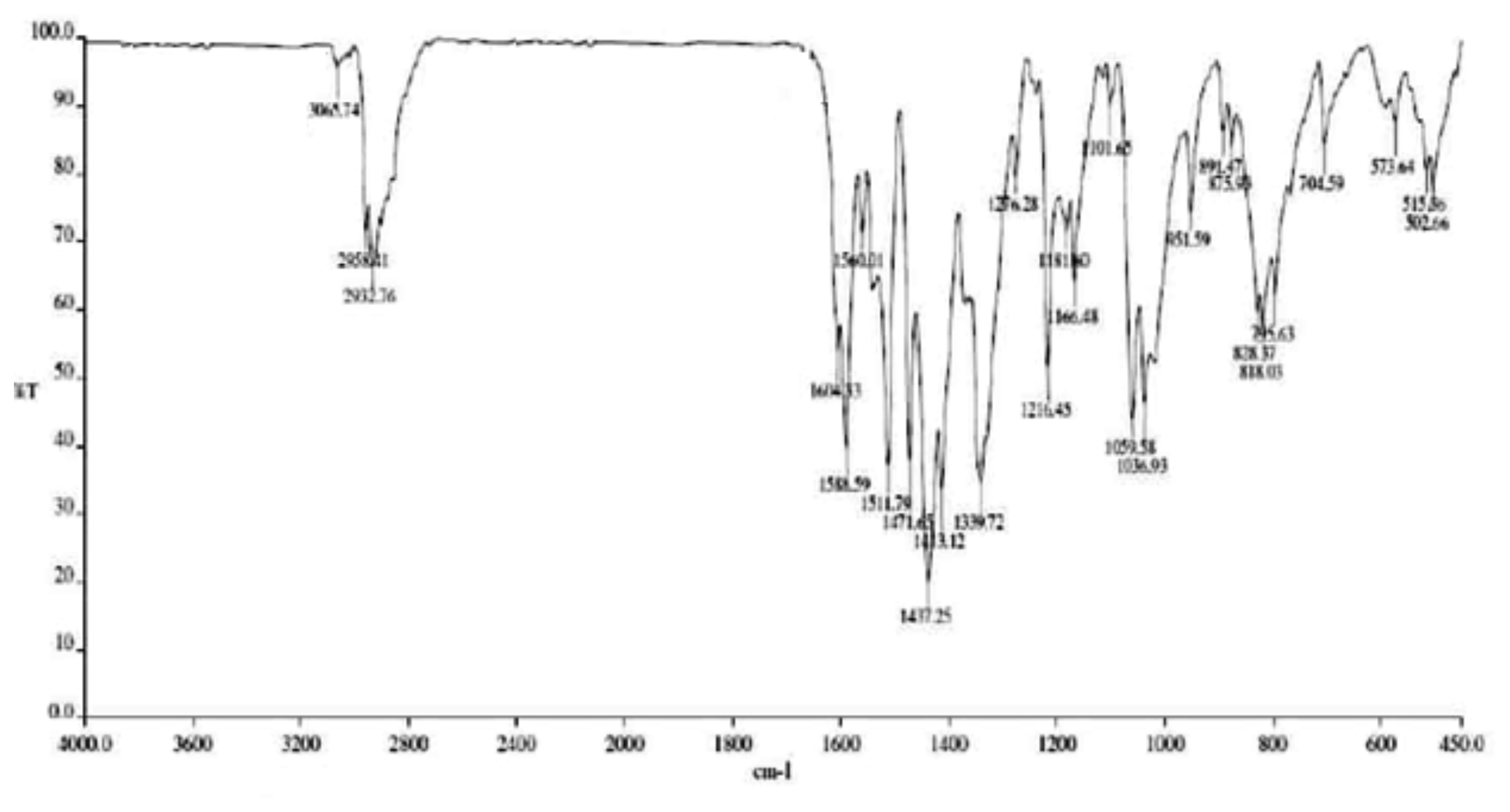

Supplement: Supplementary file 36 — IR spectrum of compound 6i [file turkjchem-46-3-766s36.tif]

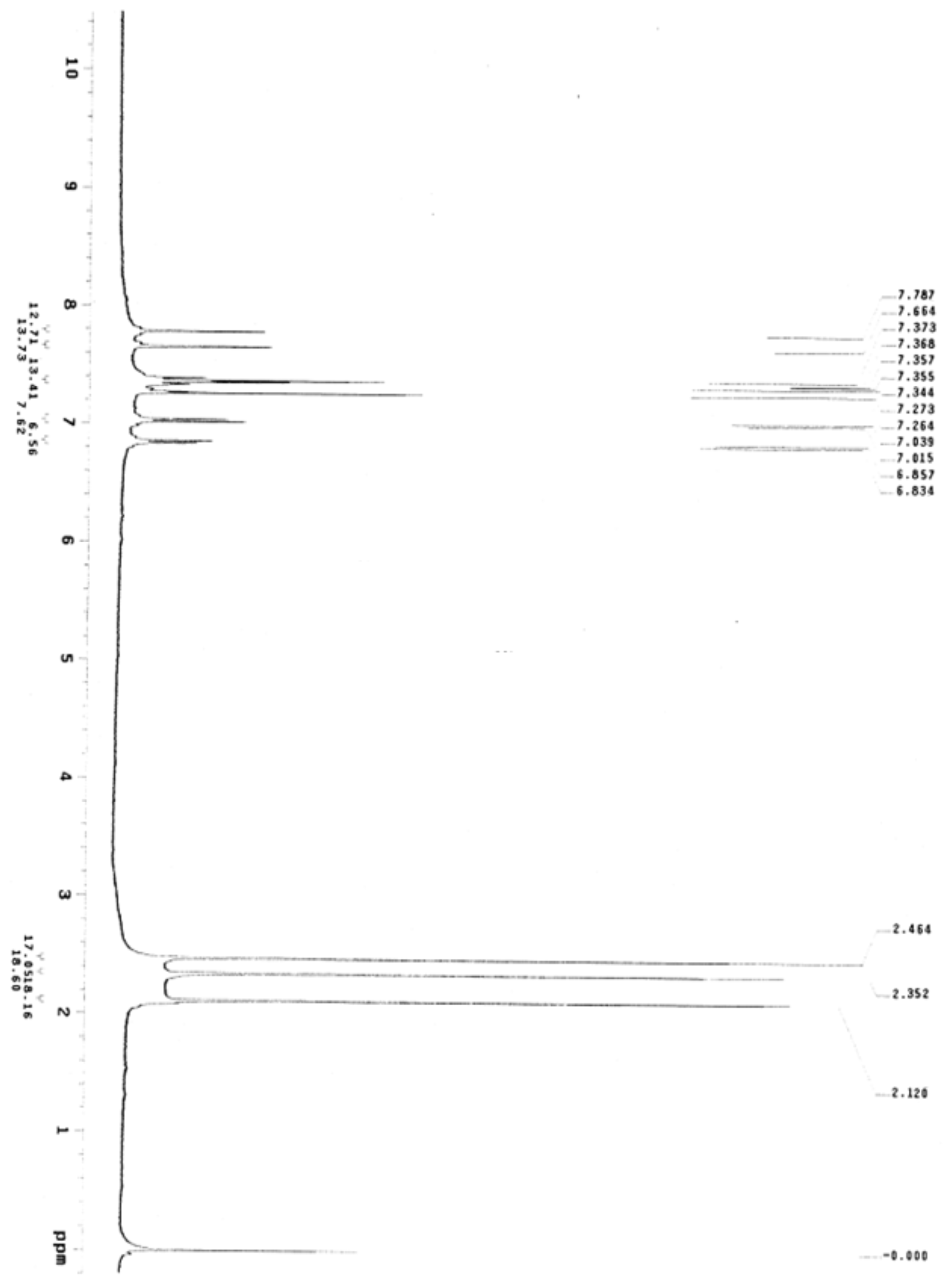

Supplement: Supplementary file 37 — 1H-NMR spectrum of compound 6j [file turkjchem-46-3-766s37.tif]

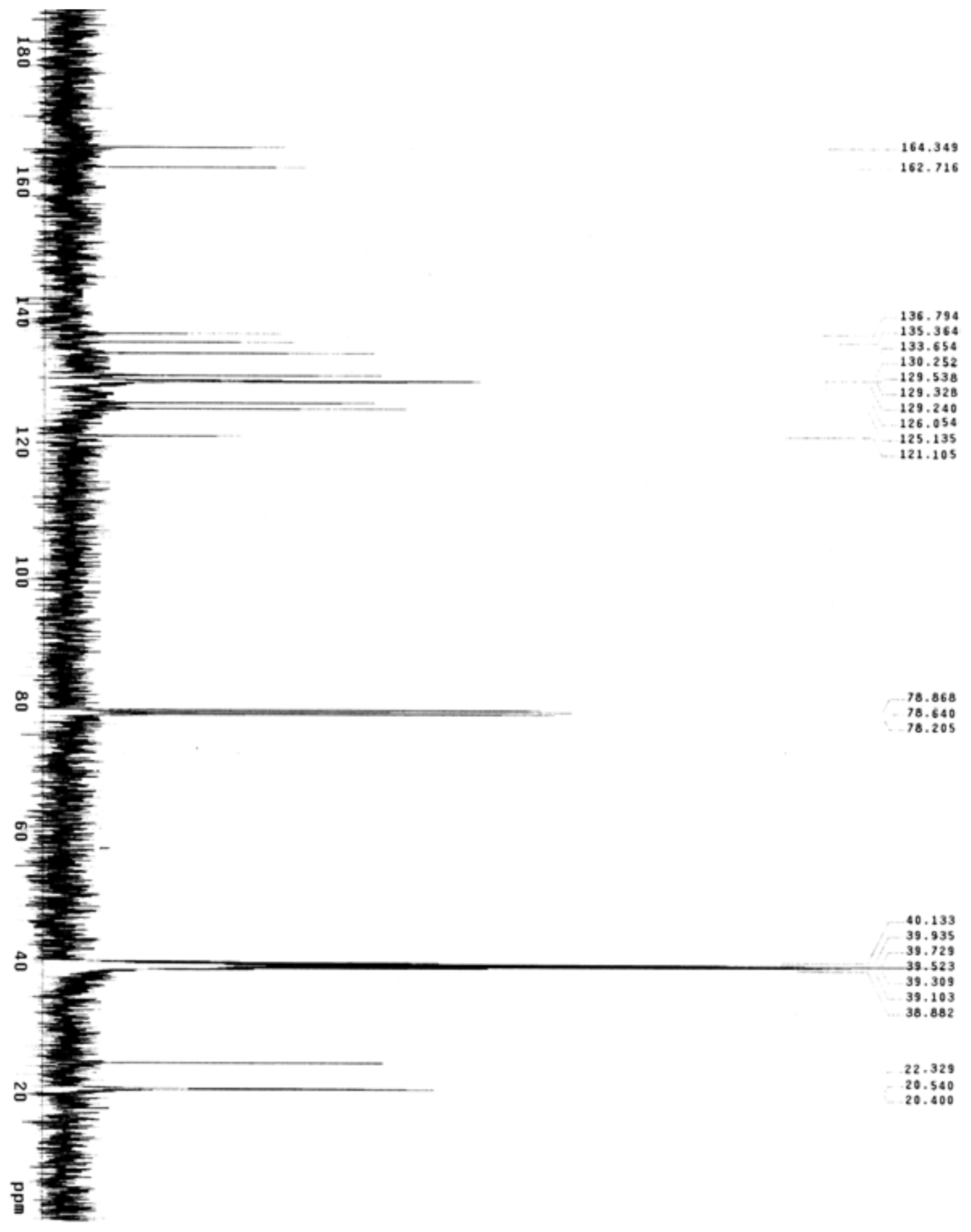

Supplement: Supplementary file 38 — 13C-NMR spectrum of compound 6j [file turkjchem-46-3-766s38.tif]

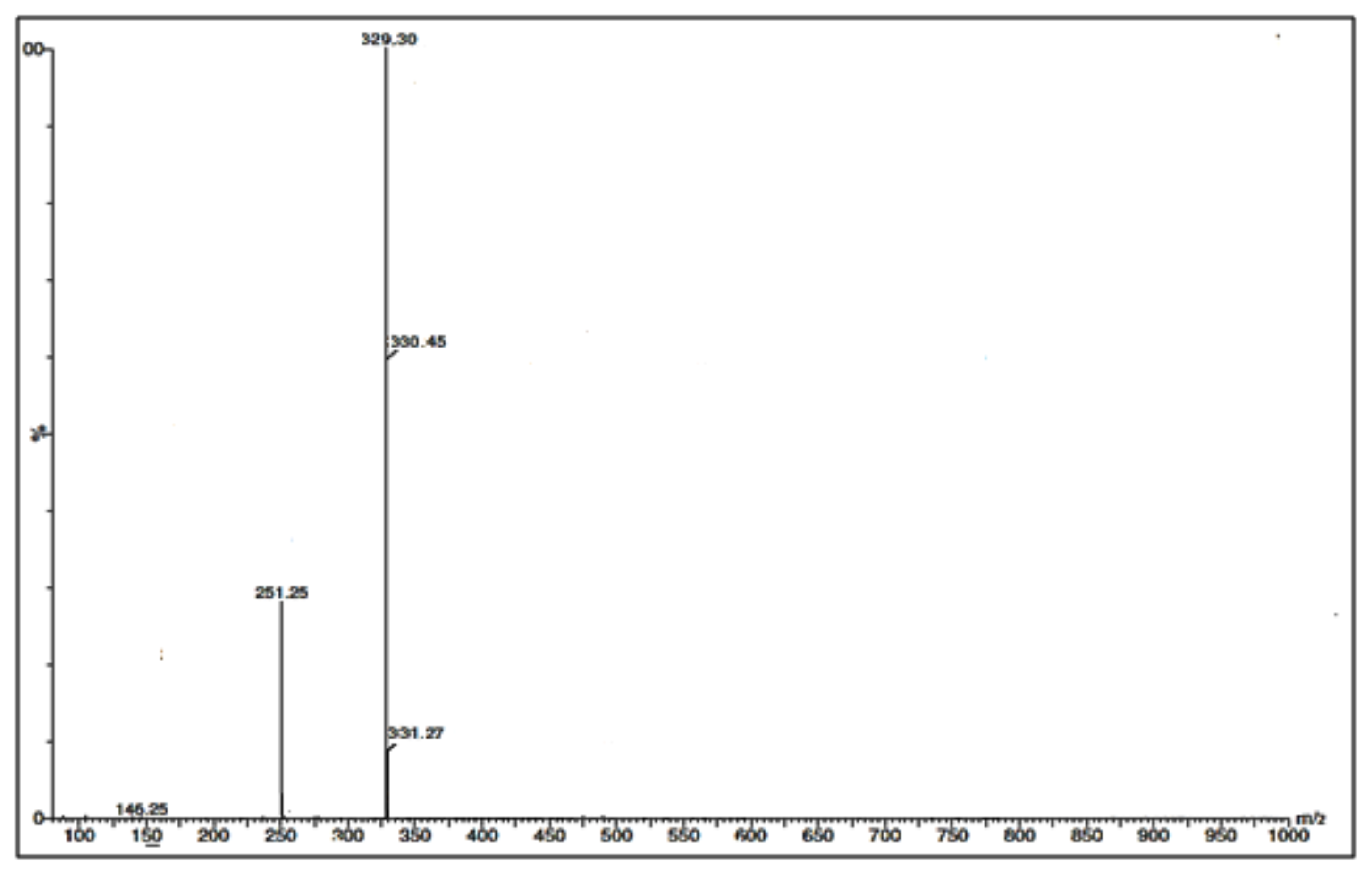

Supplement: Supplementary file 39 — Mass spectrum of compound 6j [file turkjchem-46-3-766s39.tif]

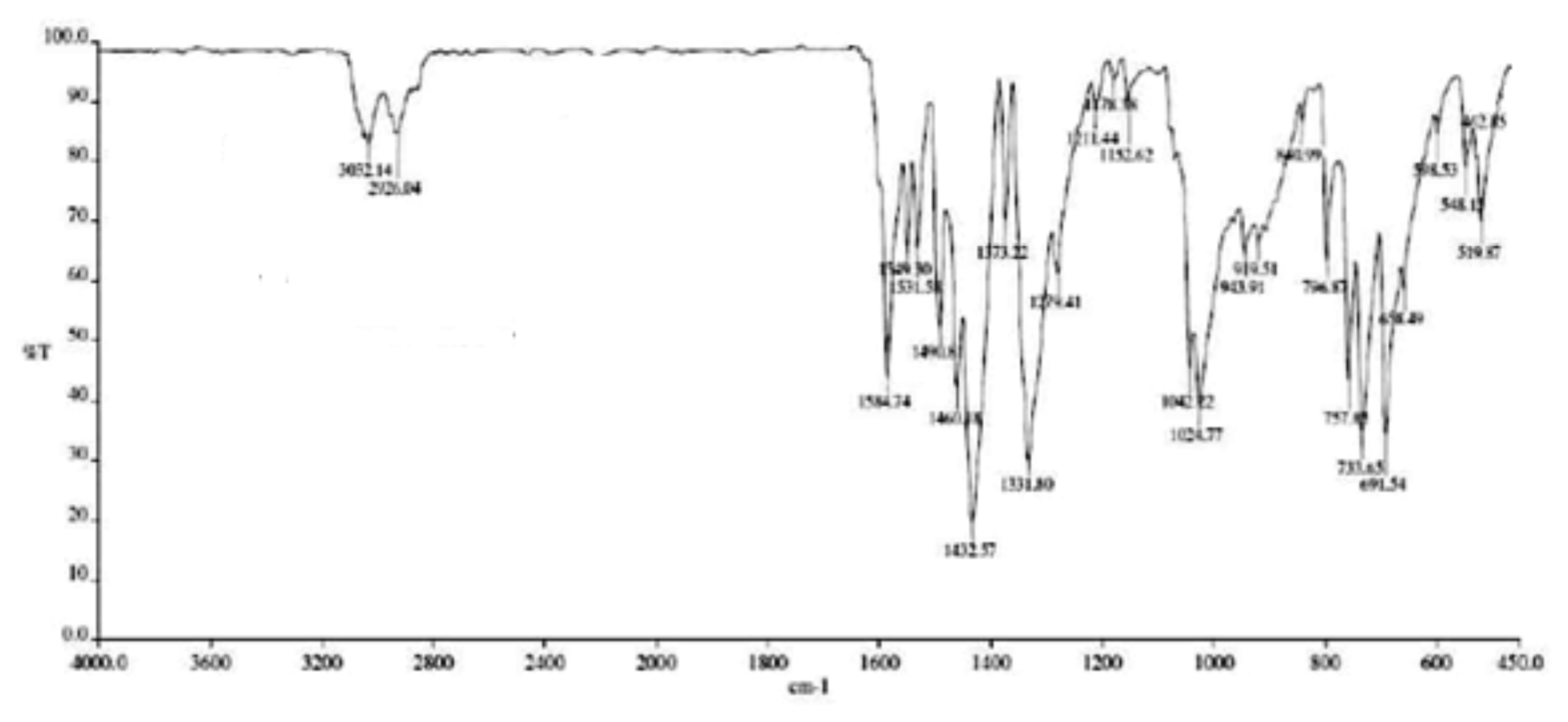

Supplement: Supplementary file 40 — IR spectrum of compound 6j [file turkjchem-46-3-766s40.tif]
